# Supplementary material for: MCMBP promotes the assembly of the MCM2–7 hetero-hexamer to ensure robust DNA replication in human cells
Source: eLife. 2022 Apr 19;11:e77393. doi: 10.7554/eLife.77393 (PMC9018068; doi:10.7554/eLife.77393)

**Figure 1a**

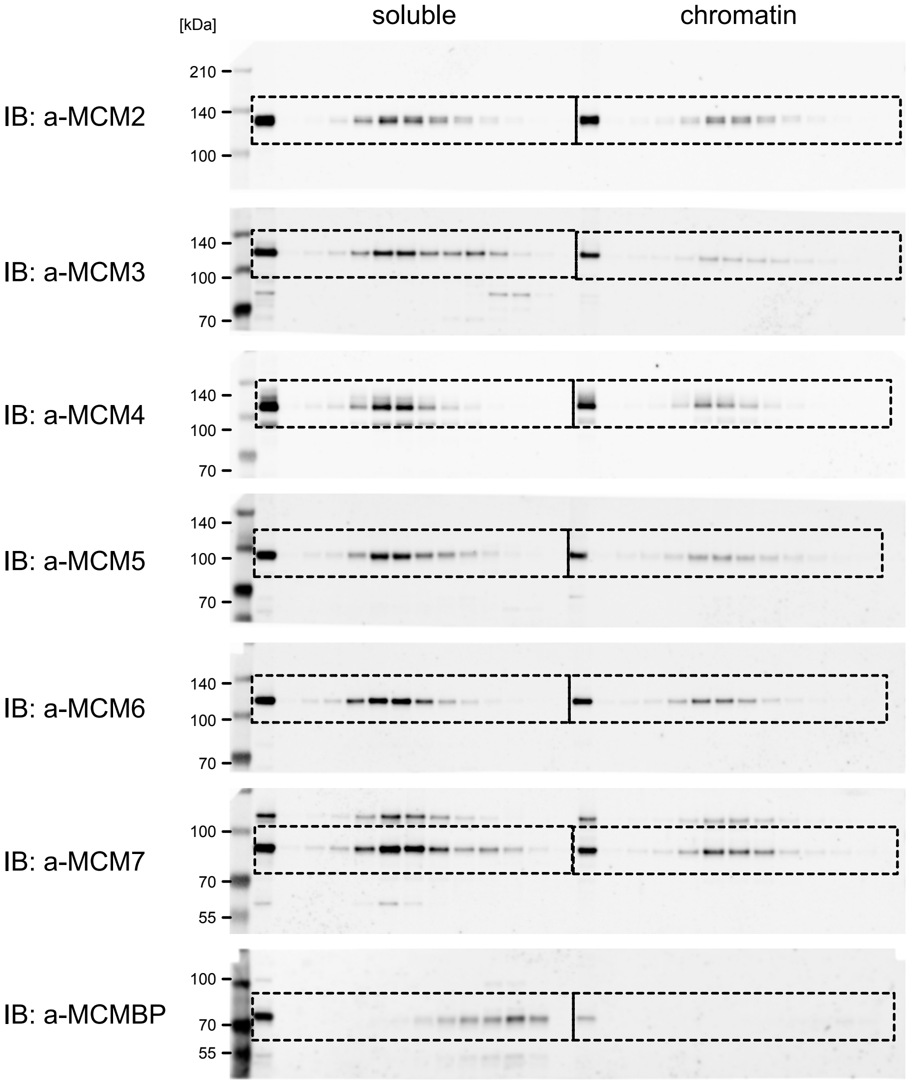


**Figure 1d**

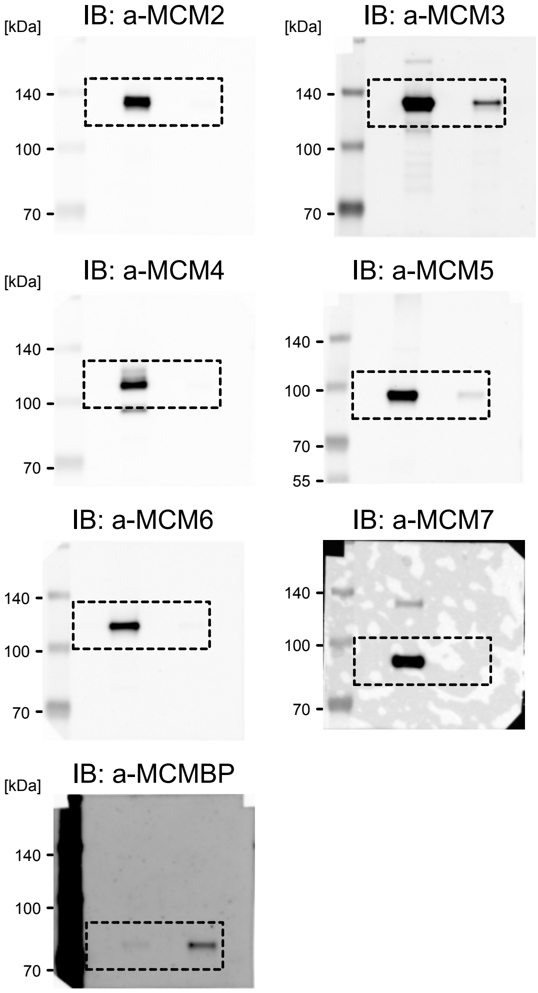


**Figure 1b**

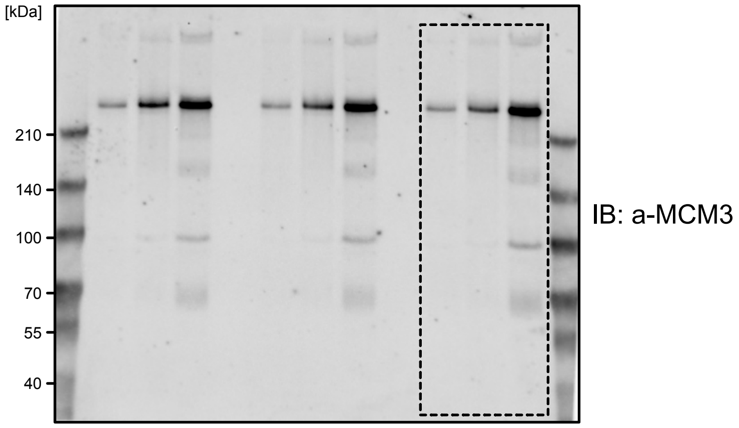

## Figure 2b

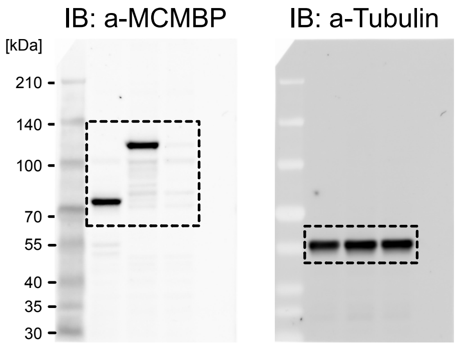


## Figure 2c

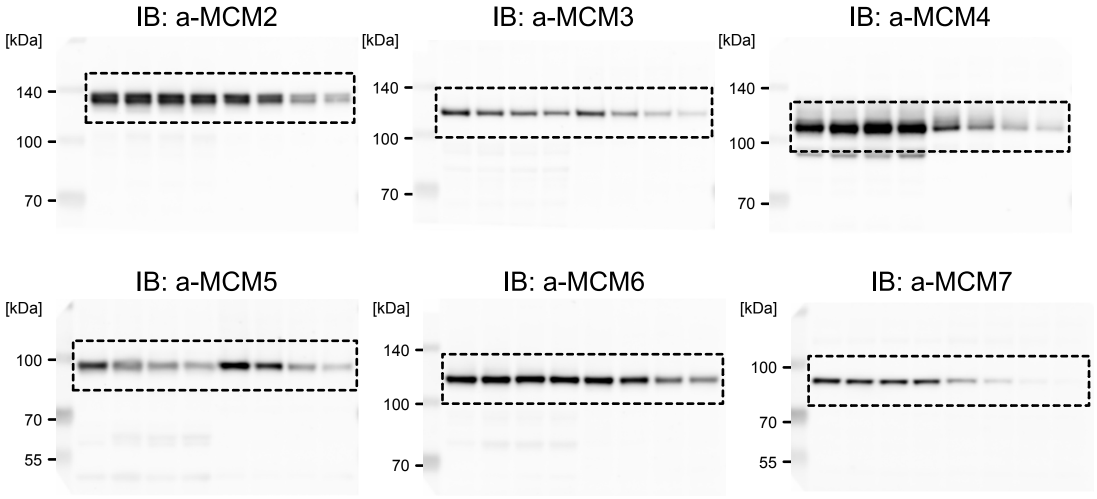


## Figure 2d

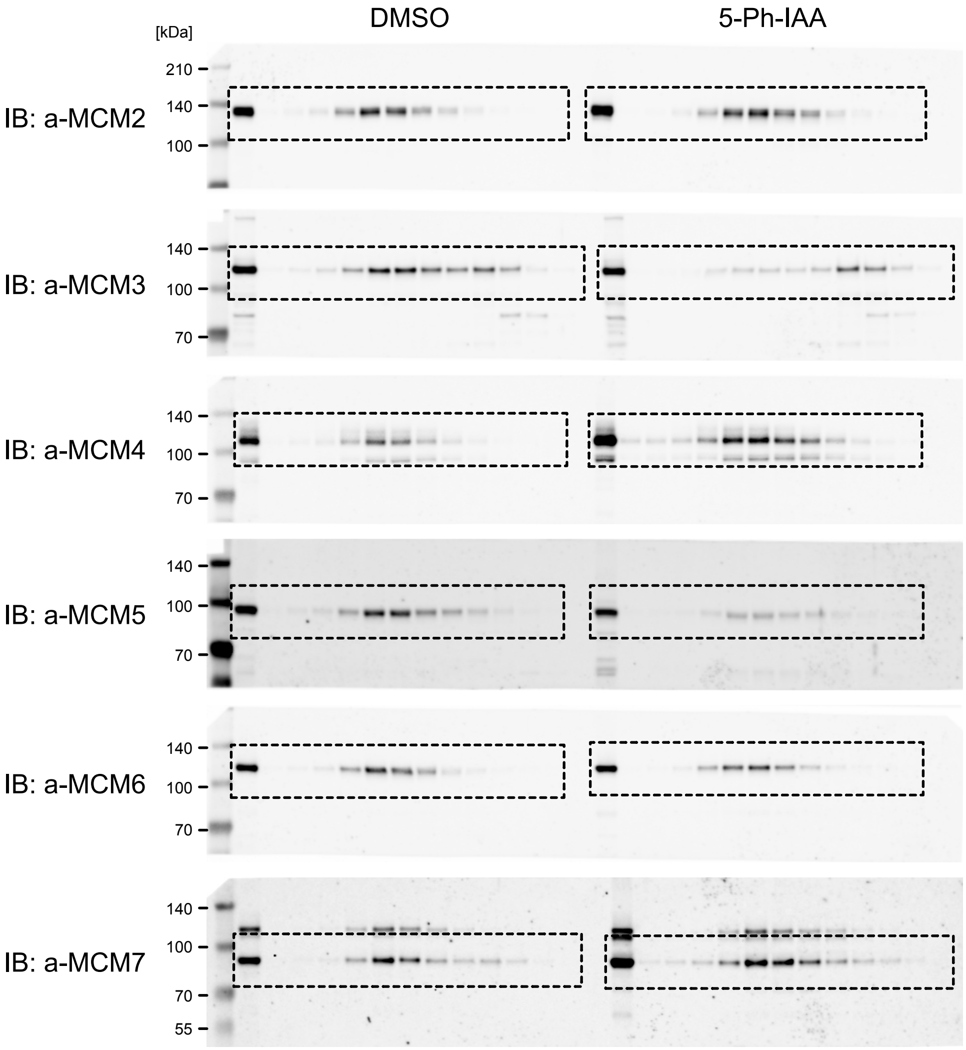

**Figure 3b**

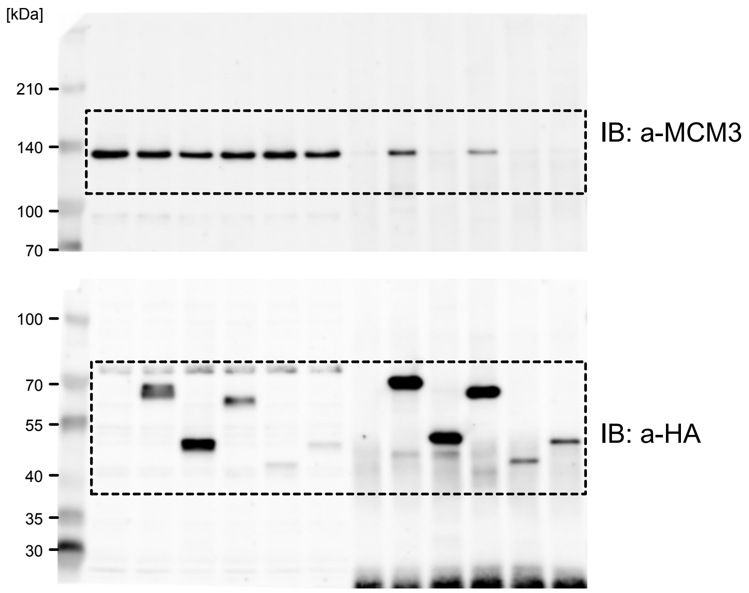


**Figure 3c**

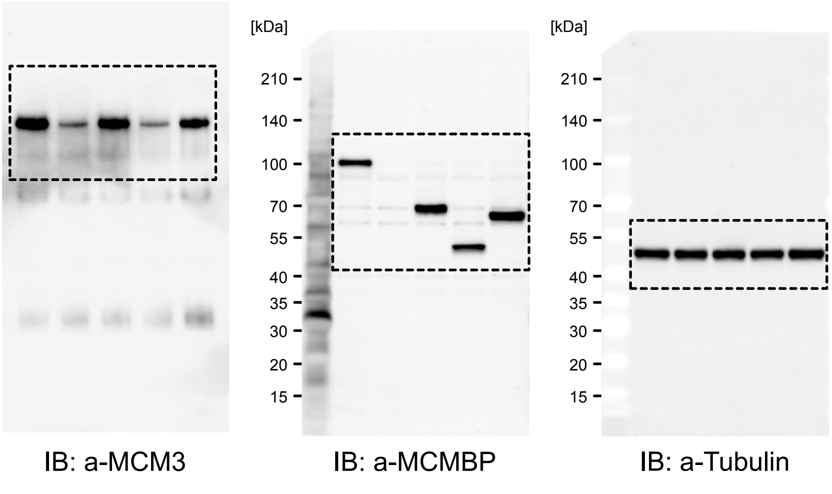

## Figure 4a

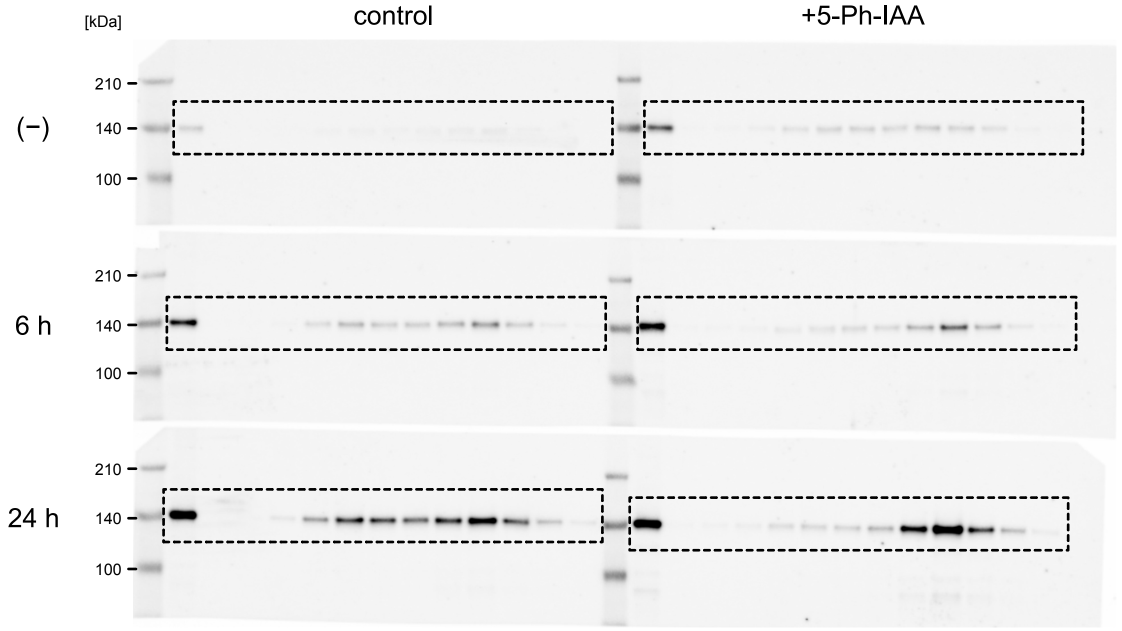


## Figure 4f

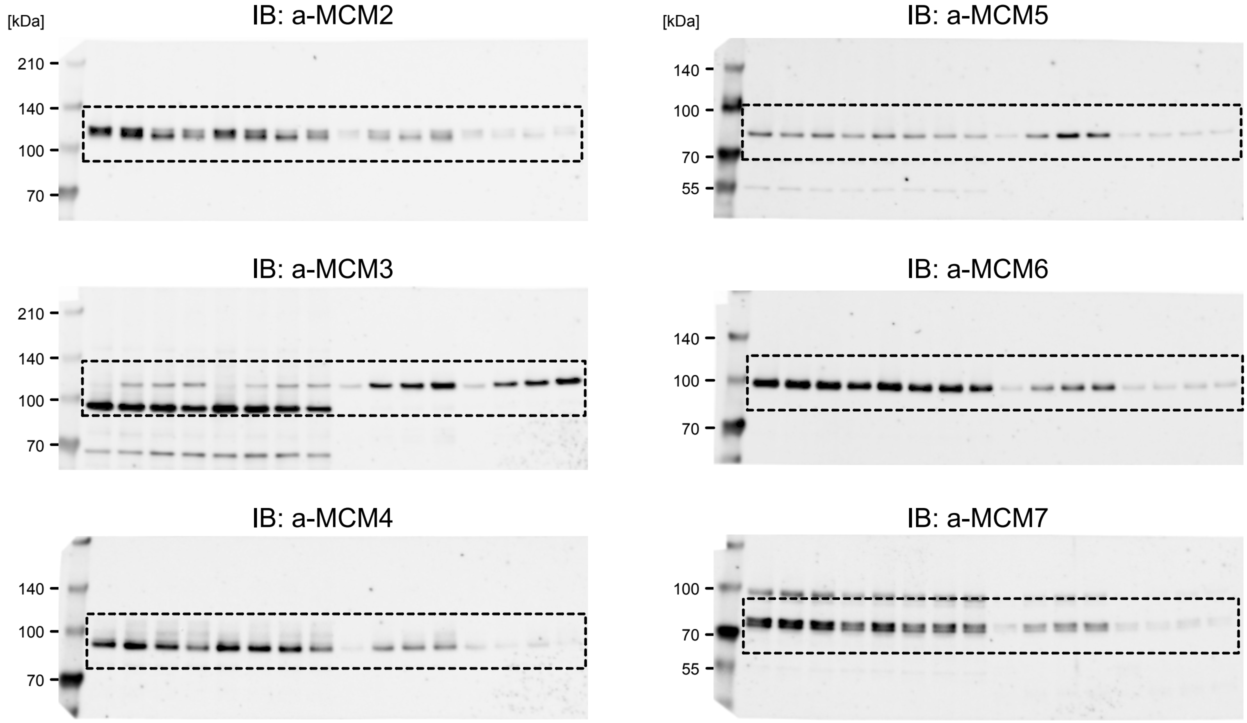

## Figure 1−figure supplement 1a

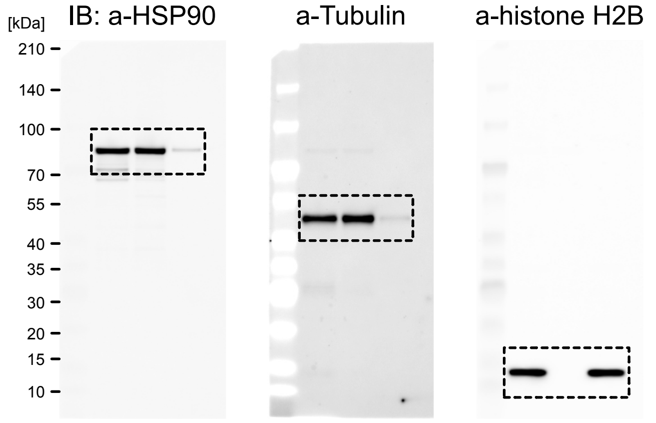


## Figure 1−figure supplement 1b

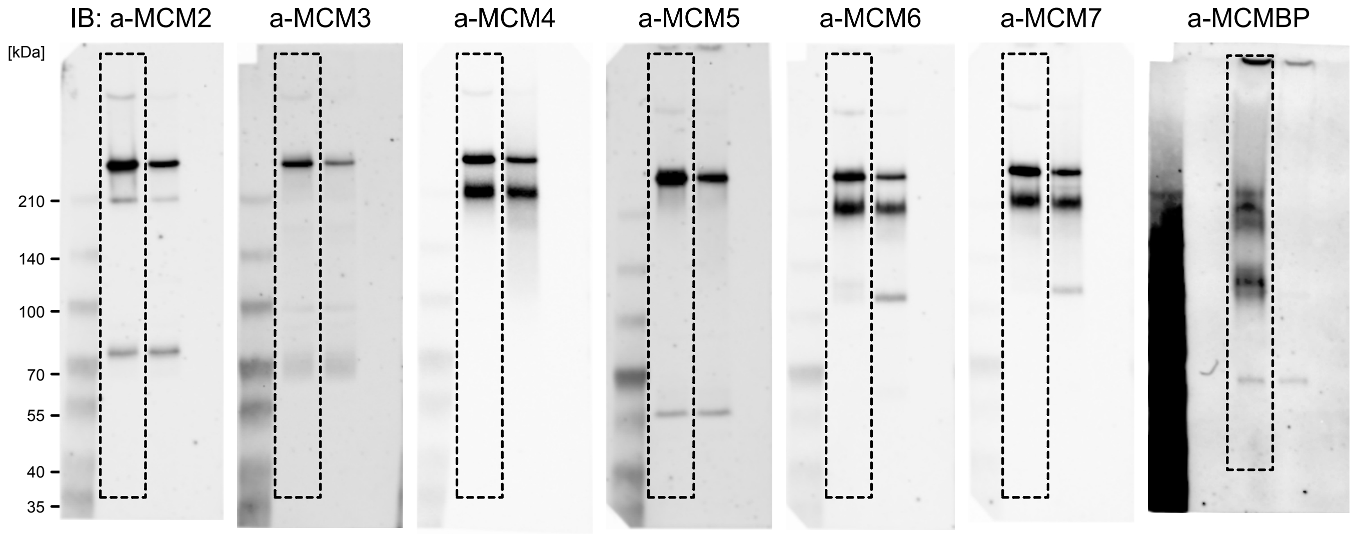


## Figure 1−figure supplement 1c

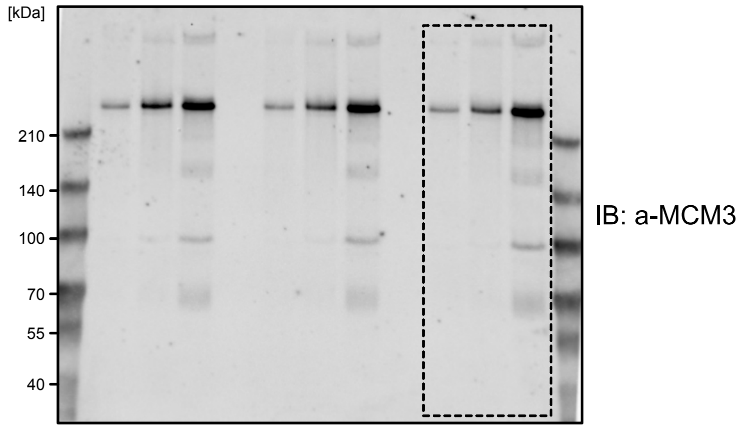

**Figure 1−figure supplement 1d**

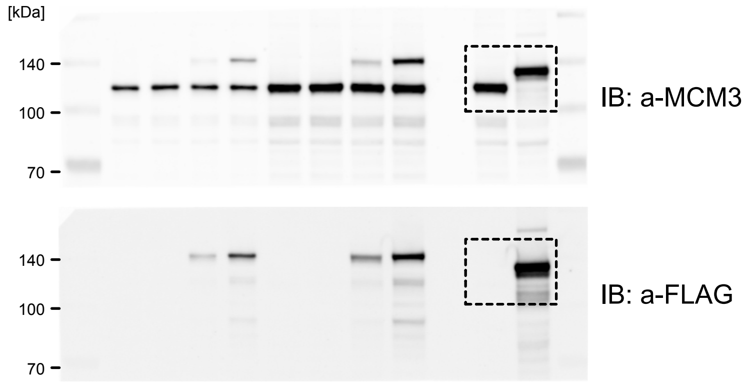


**Figure 1−figure supplement 1e**

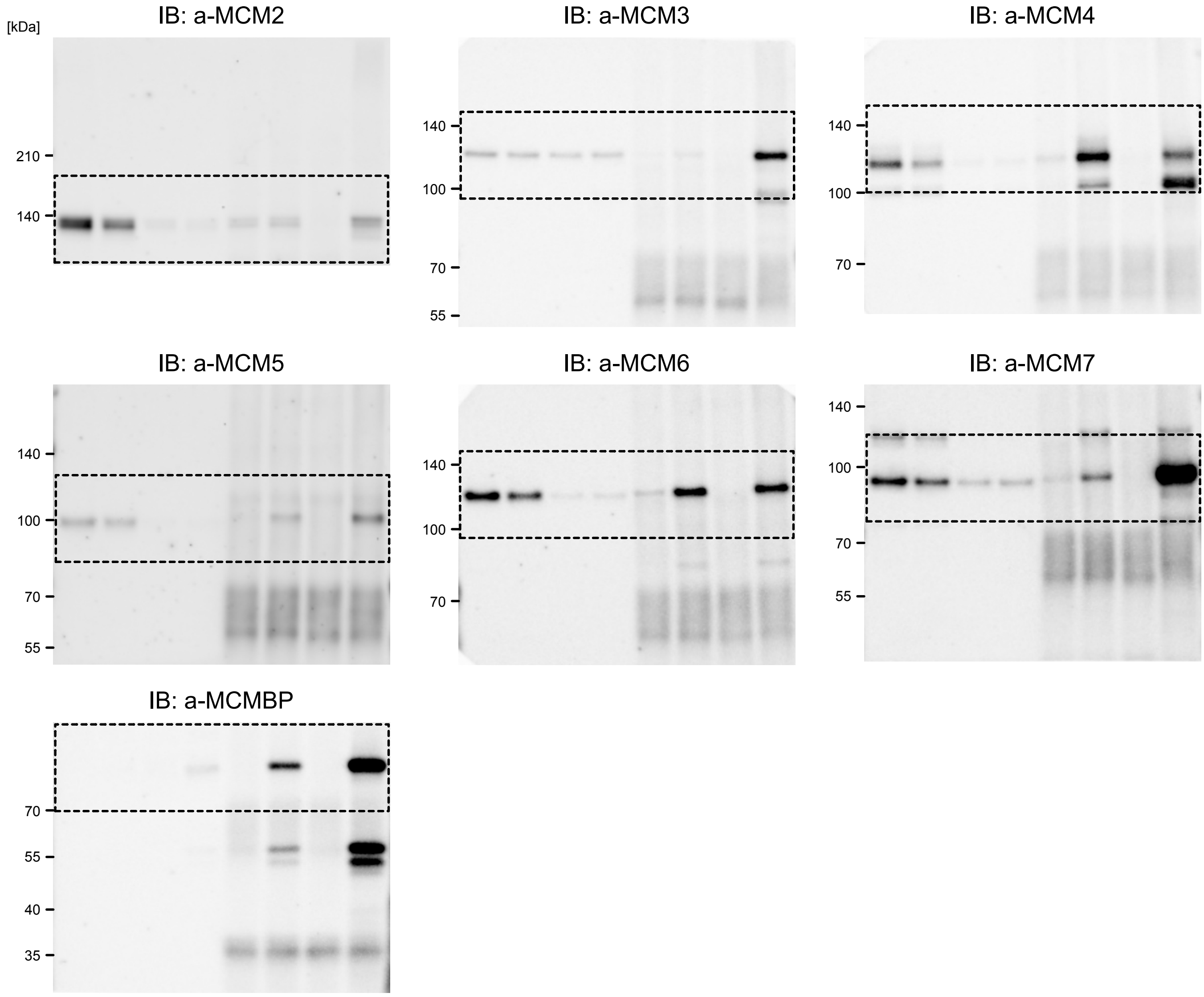

# Figure 2−figure supplement 1c

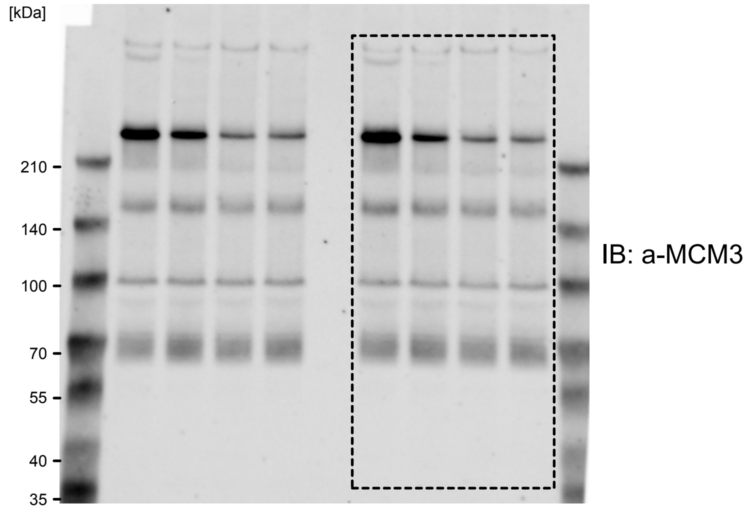

## Figure 3−figure supplement 1b

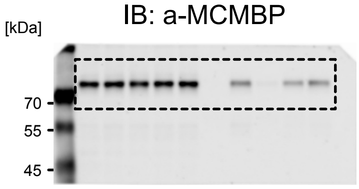


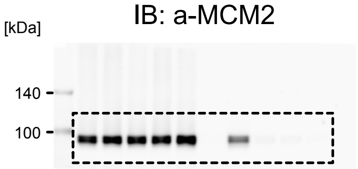


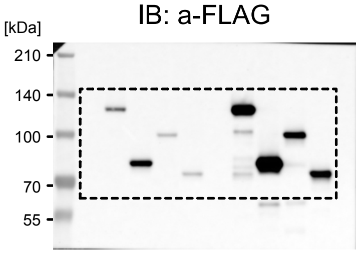

## Figure 4−figure supplement 1b

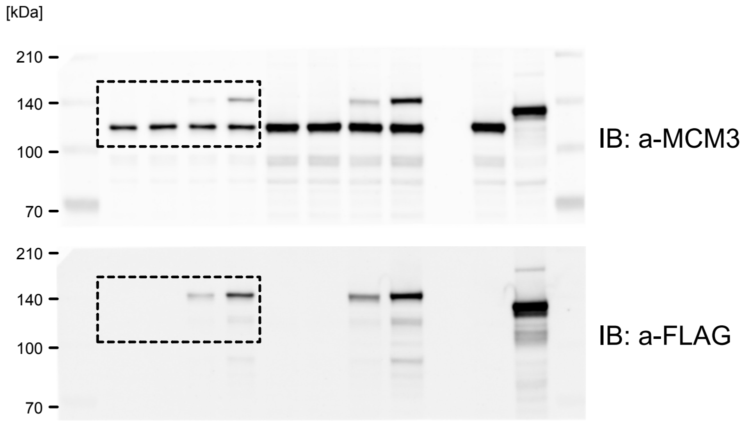


## Figure 4−figure supplement 1e

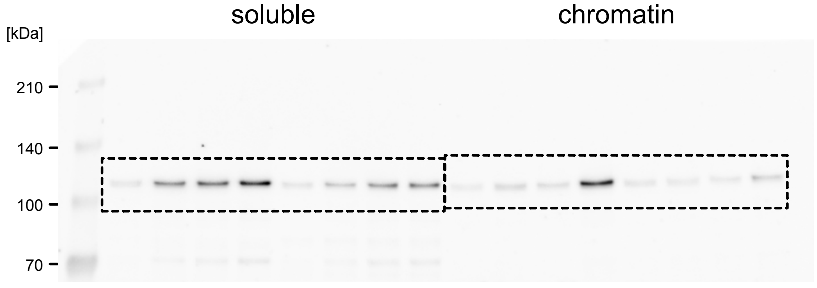

## Figure 4−figure supplement 2a

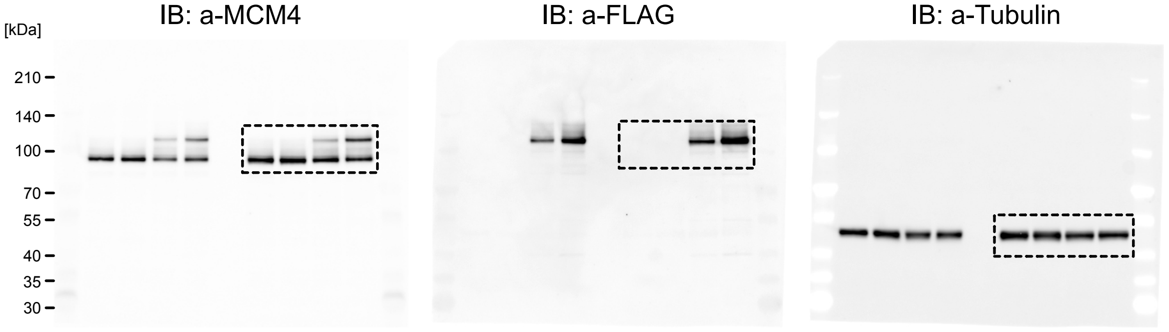


## Figure 4−figure supplement 2d

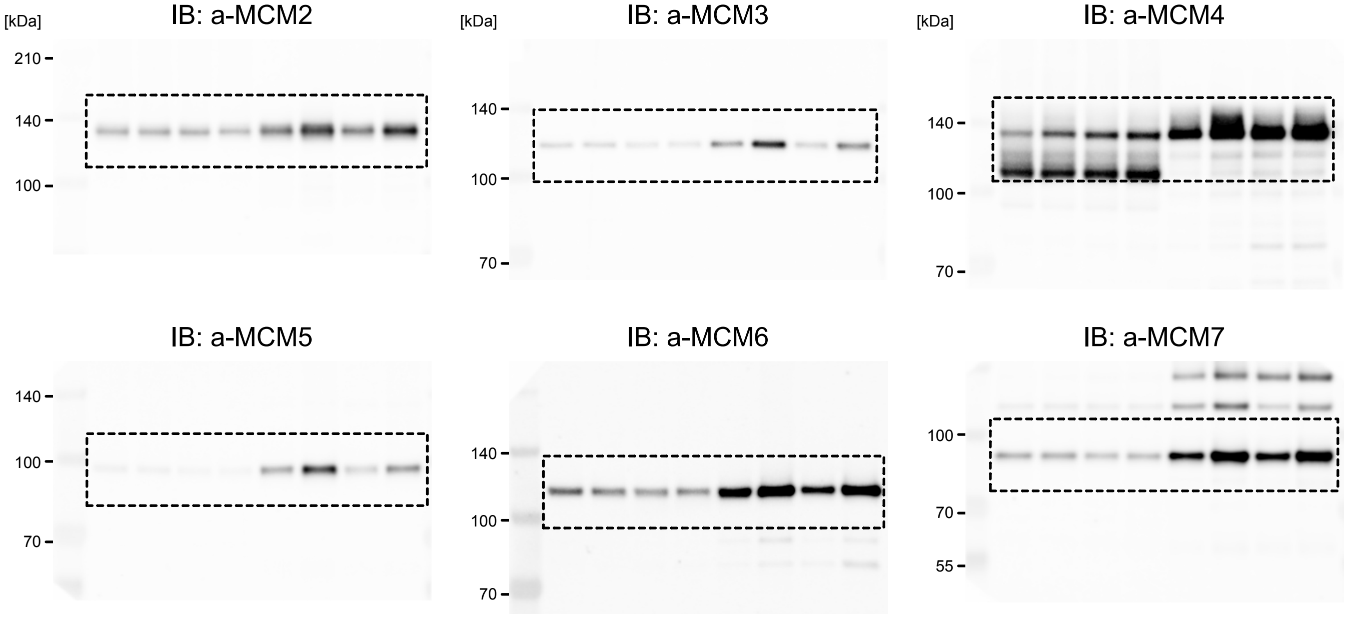

## Figure 5–figure supplement 1c

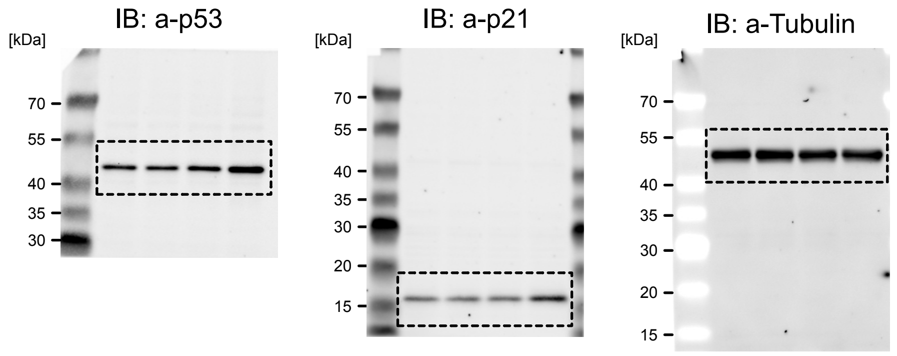


## Figure 5–figure supplement 1e

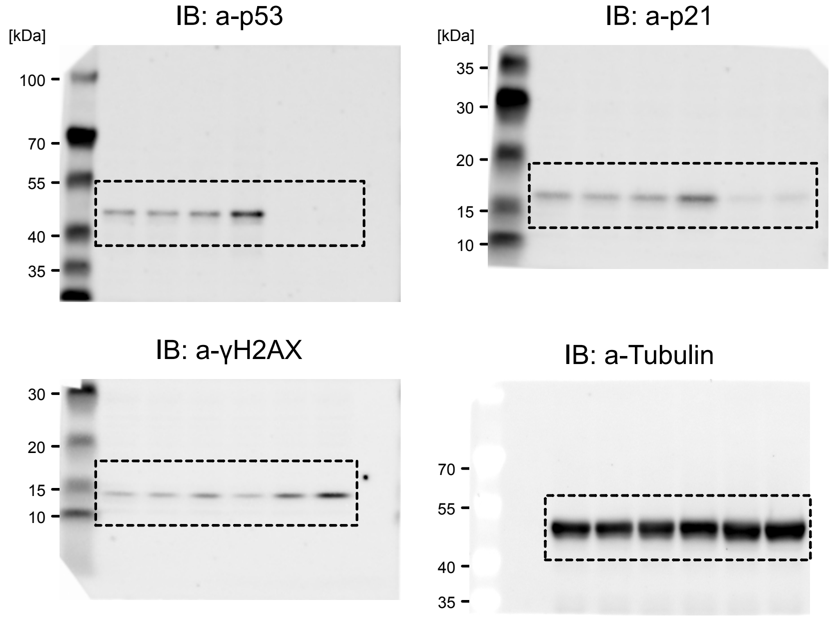

Supplement: Source data 2. [file elife-77393-data2.zip › Source data 2/WB data.pdf]
