# Supplementary figures and images for: MCMBP promotes the assembly of the MCM2–7 hetero-hexamer to ensure robust DNA replication in human cells
Source: eLife. 2022 Apr 19;11:e77393. doi: 10.7554/eLife.77393 (PMC9018068; doi:10.7554/eLife.77393)

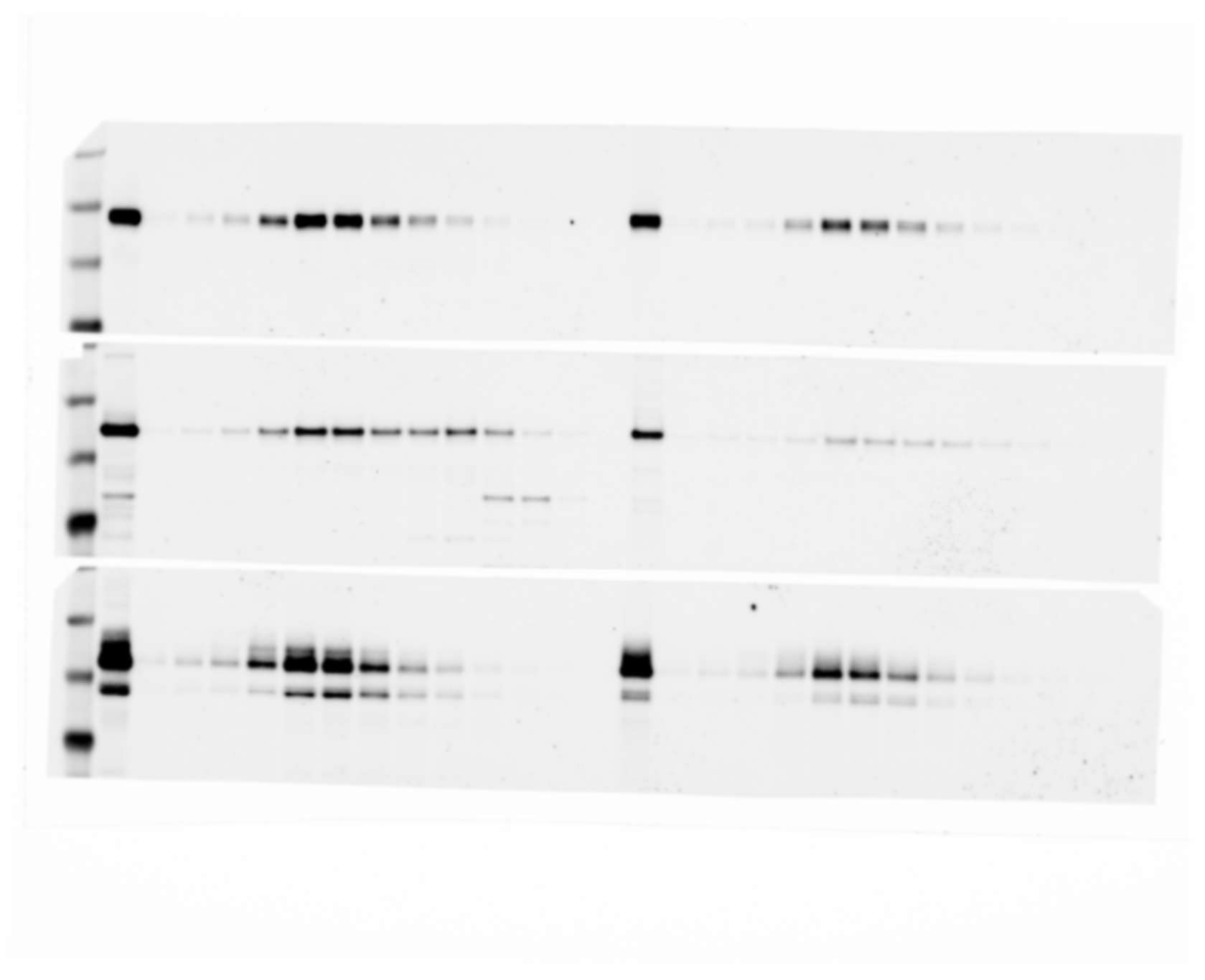

Supplement: Source data 2. [file elife-77393-data2.zip › Source data 2/WB raw data/Figure 1a/MCM3.tif]

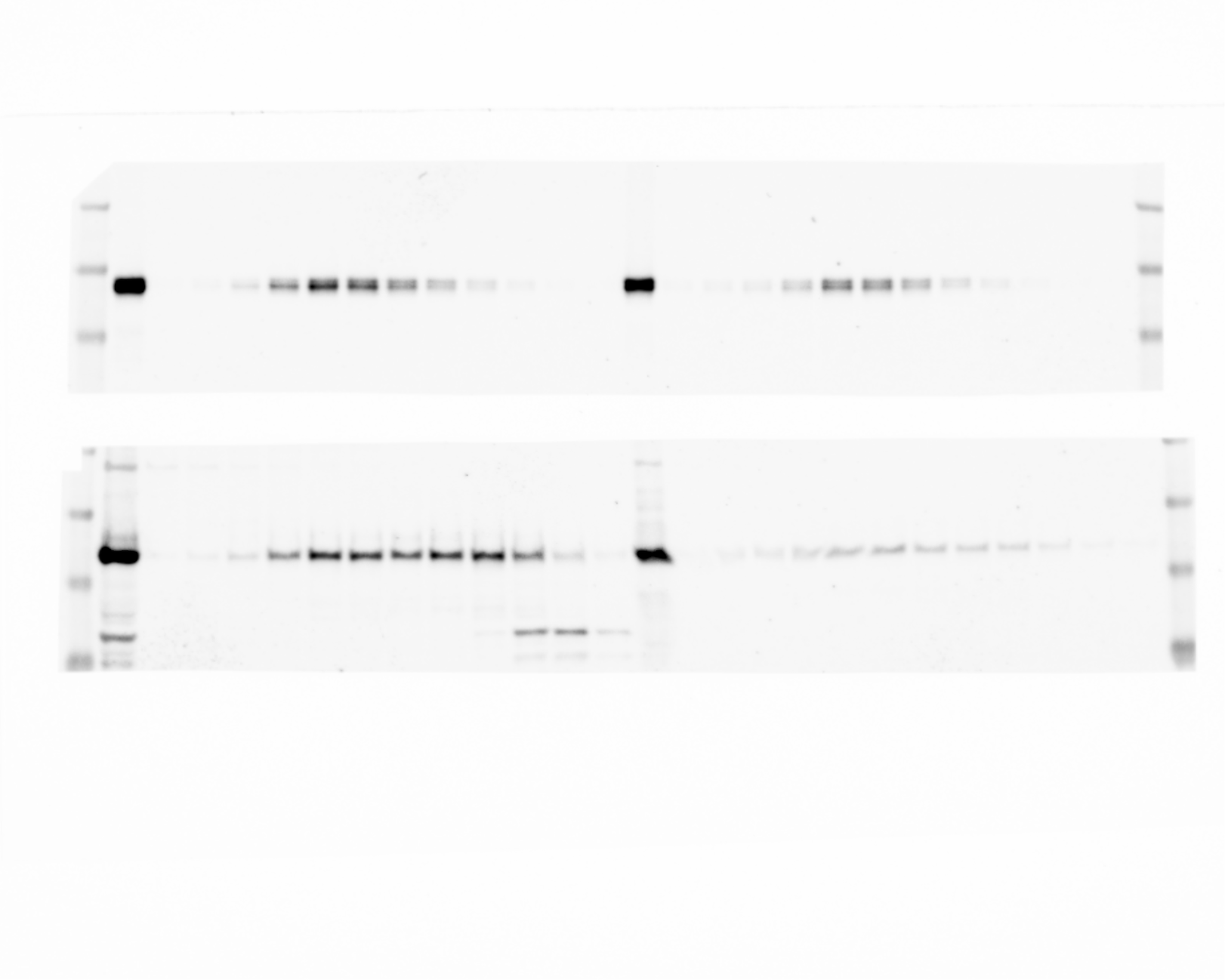

Supplement: Source data 2. [file elife-77393-data2.zip › Source data 2/WB raw data/Figure 1a/MCM2.tif]

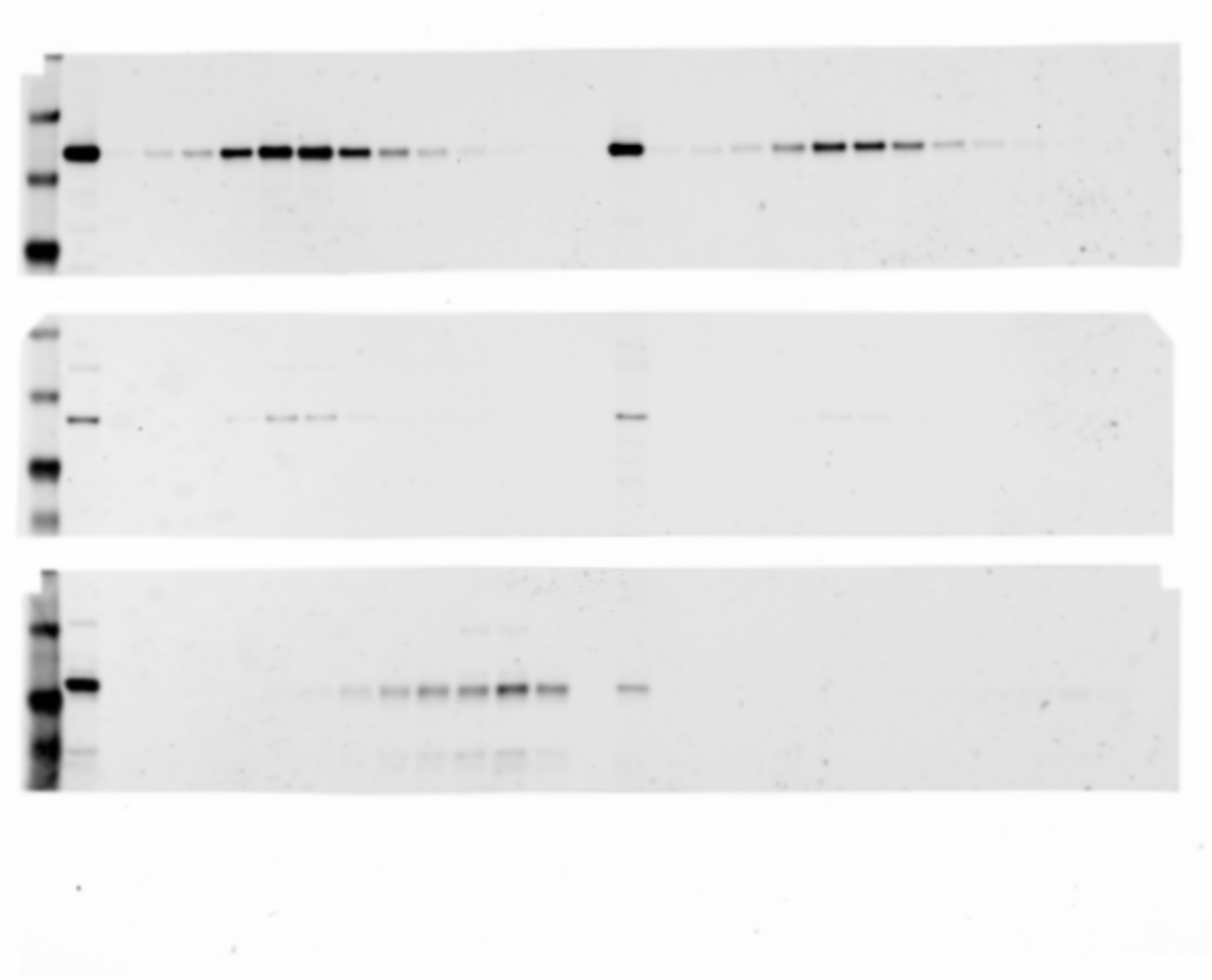

Supplement: Source data 2. [file elife-77393-data2.zip › Source data 2/WB raw data/Figure 1a/MCMBP.tif]

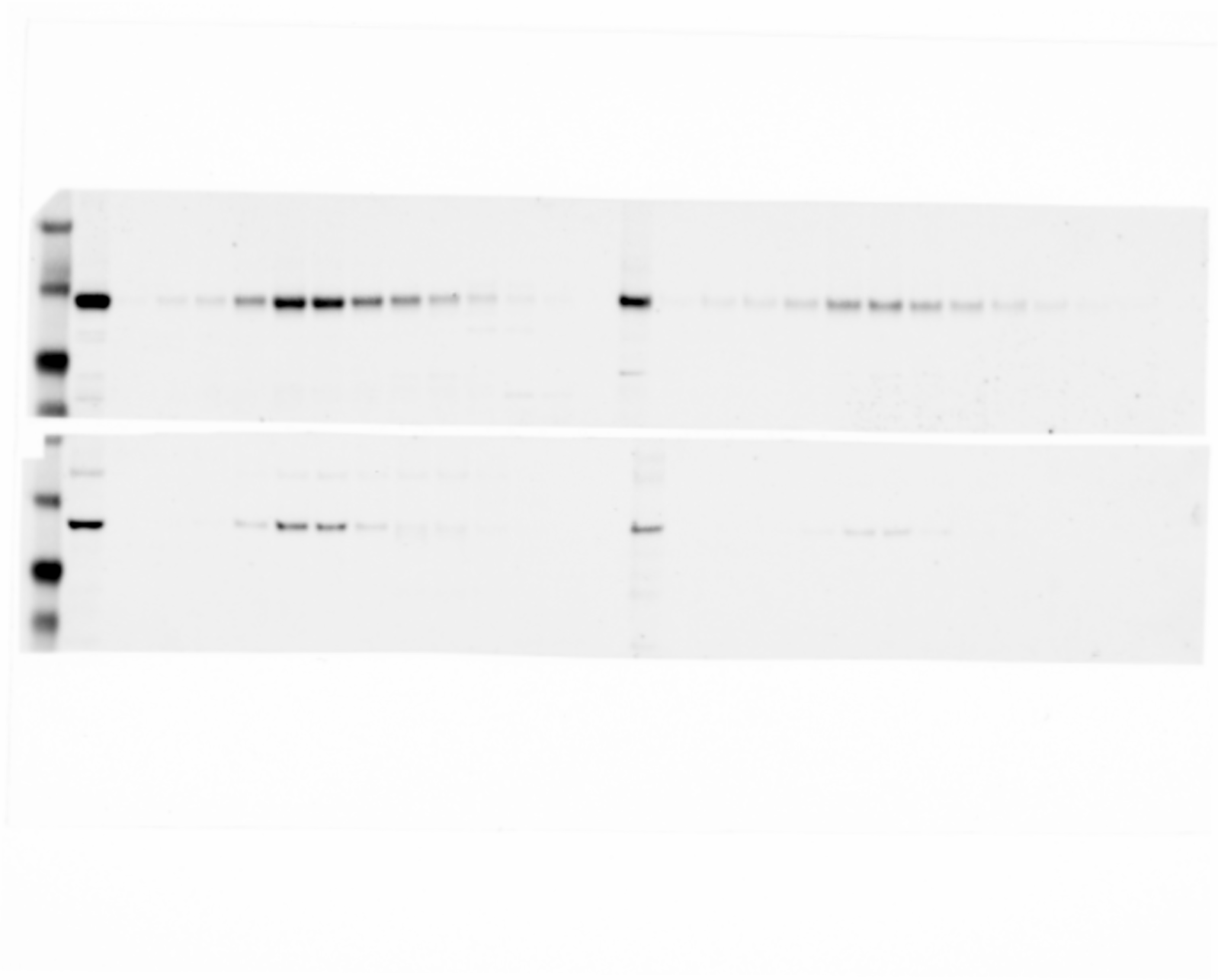

Supplement: Source data 2. [file elife-77393-data2.zip › Source data 2/WB raw data/Figure 1a/MCM5.tif]

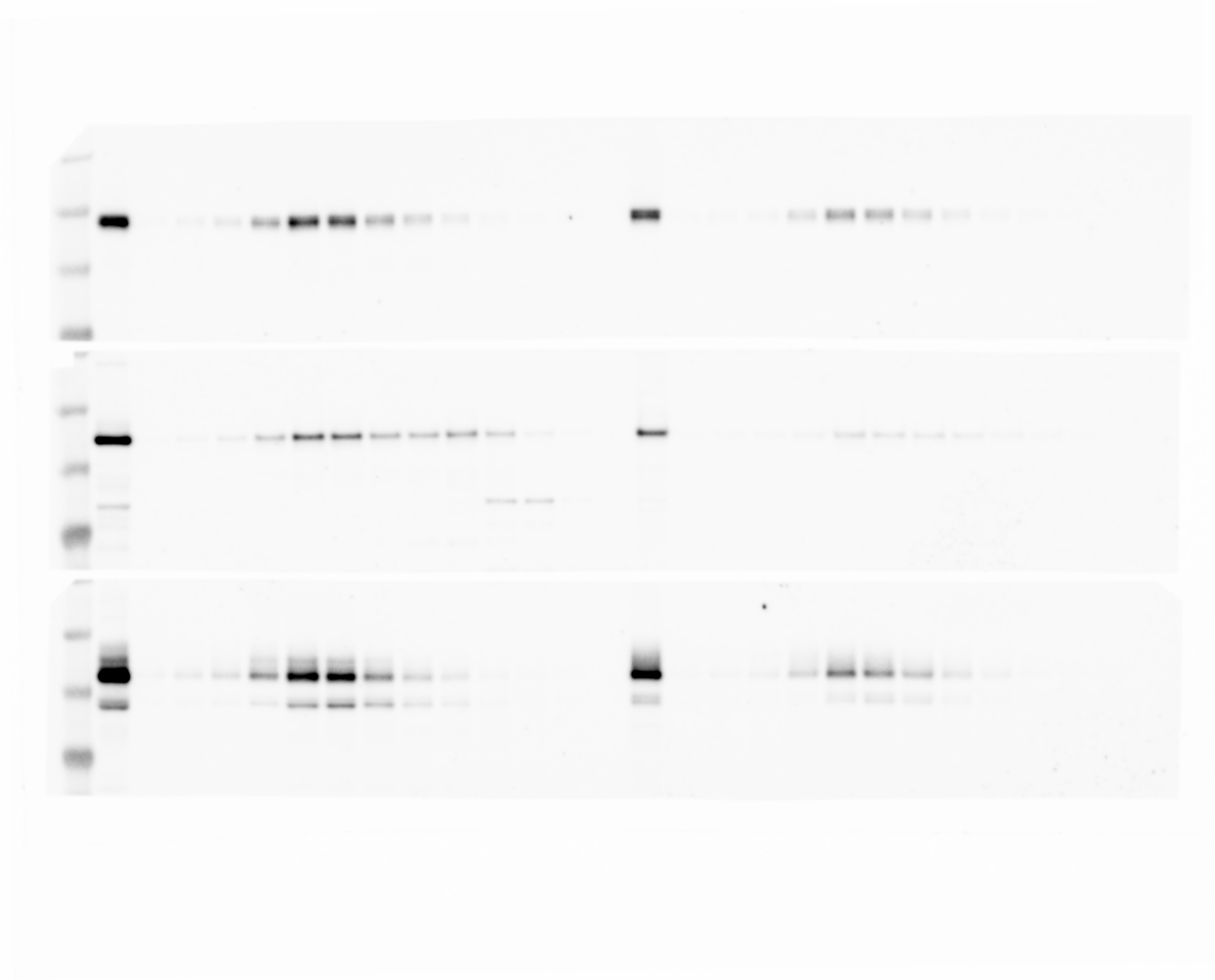

Supplement: Source data 2. [file elife-77393-data2.zip › Source data 2/WB raw data/Figure 1a/MCM4.tif]

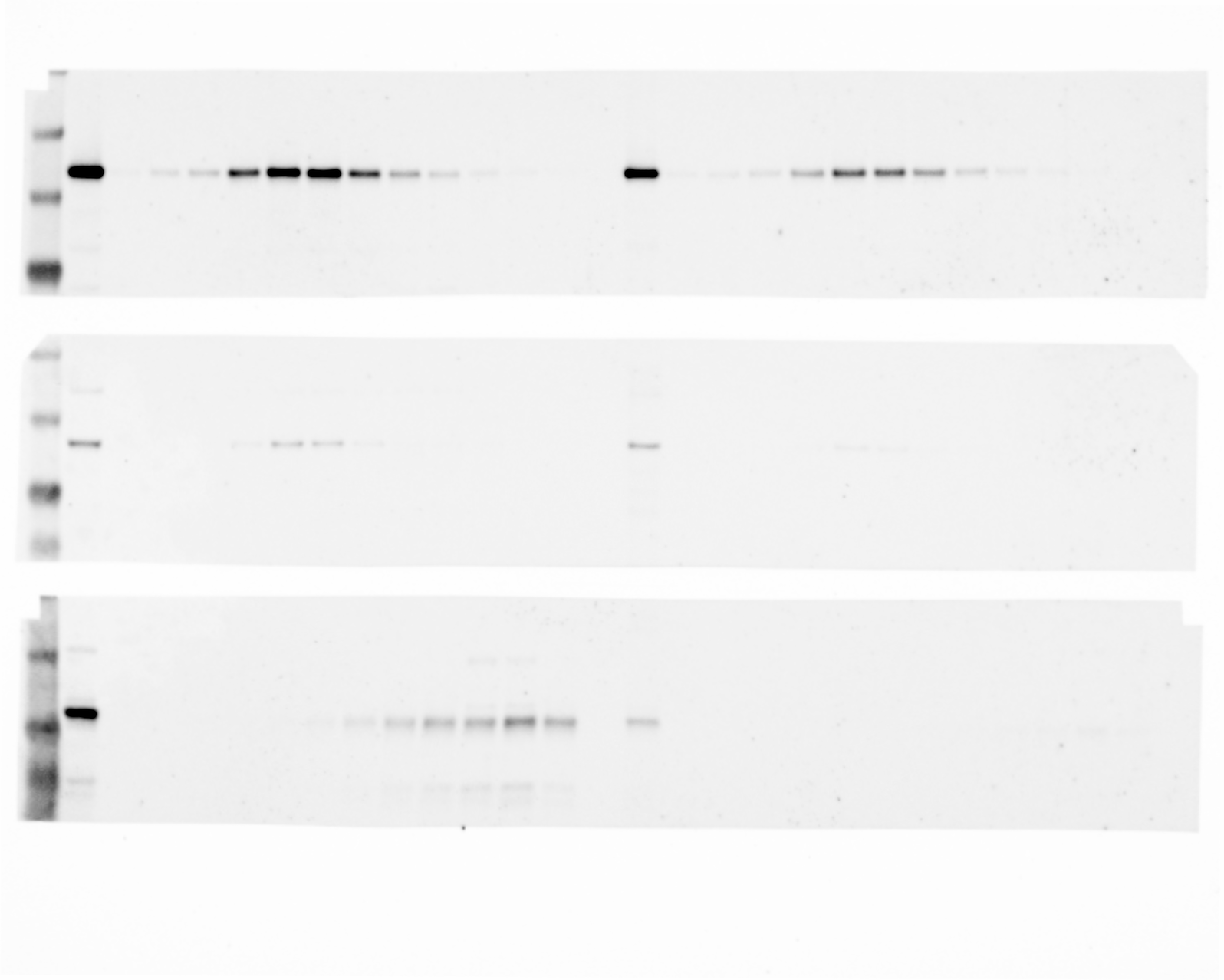

Supplement: Source data 2. [file elife-77393-data2.zip › Source data 2/WB raw data/Figure 1a/MCM6.tif]

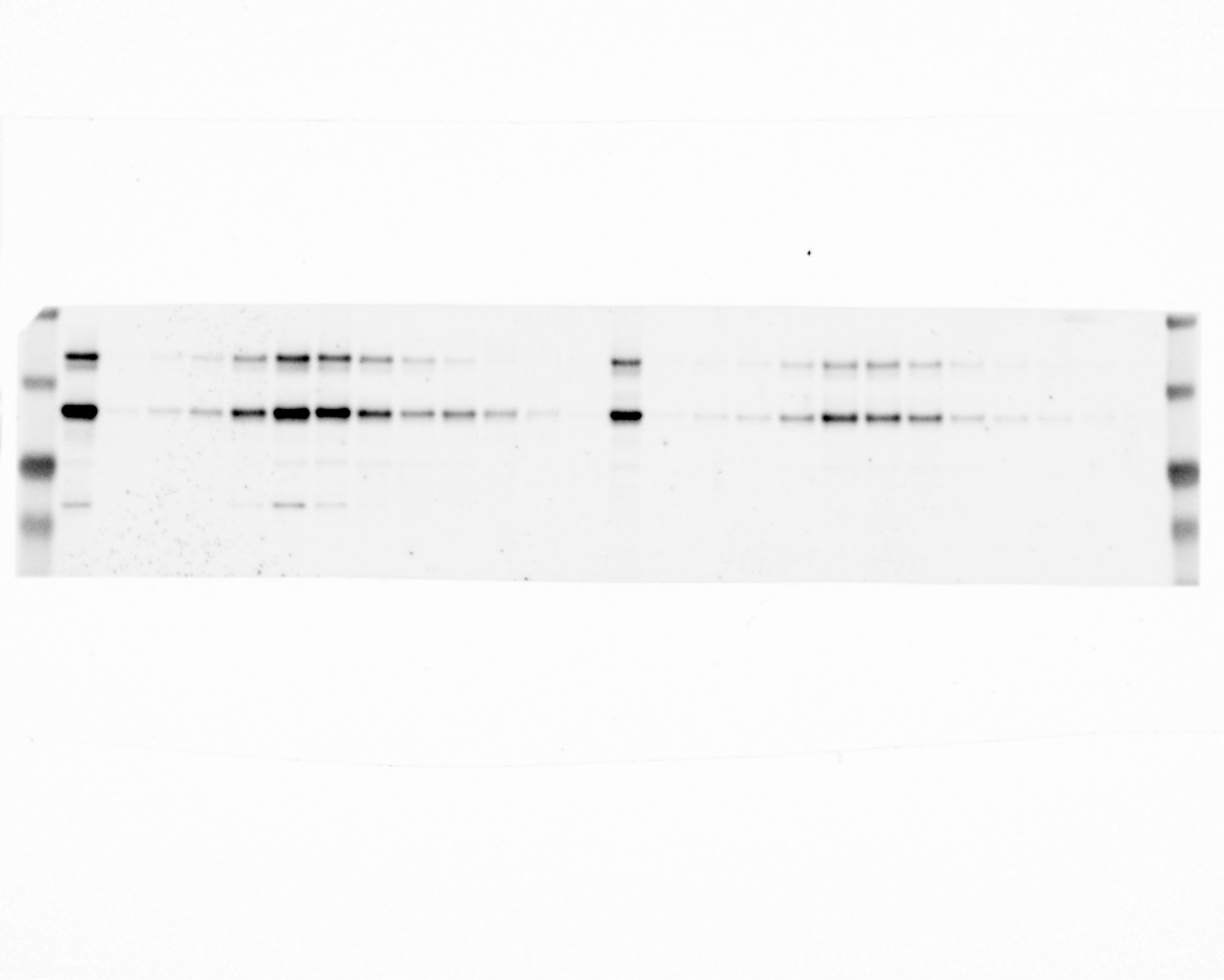

Supplement: Source data 2. [file elife-77393-data2.zip › Source data 2/WB raw data/Figure 1a/MCM7.tif]

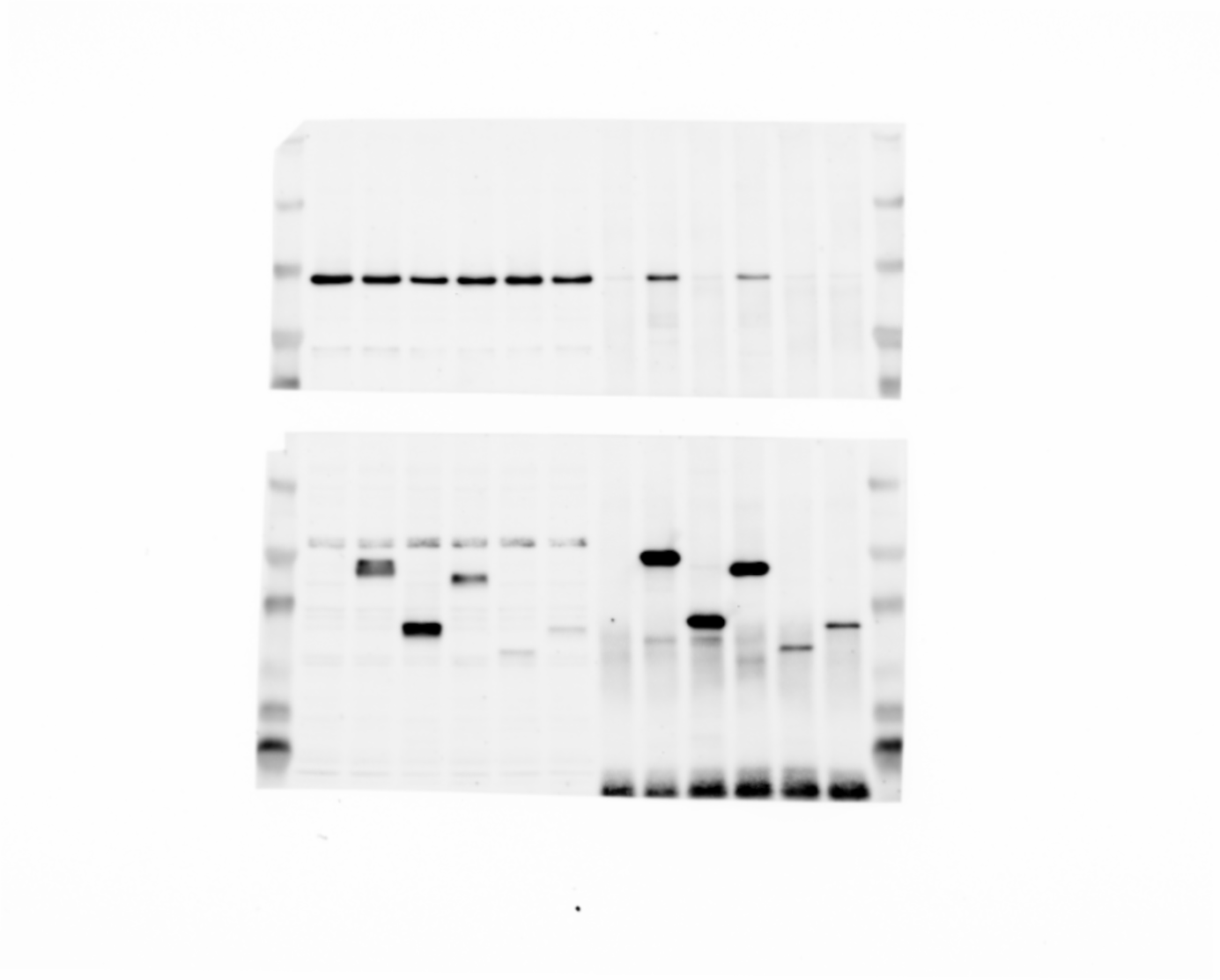

Supplement: Source data 2. [file elife-77393-data2.zip › Source data 2/WB raw data/Figure 3b/MCM3, HA.tif]

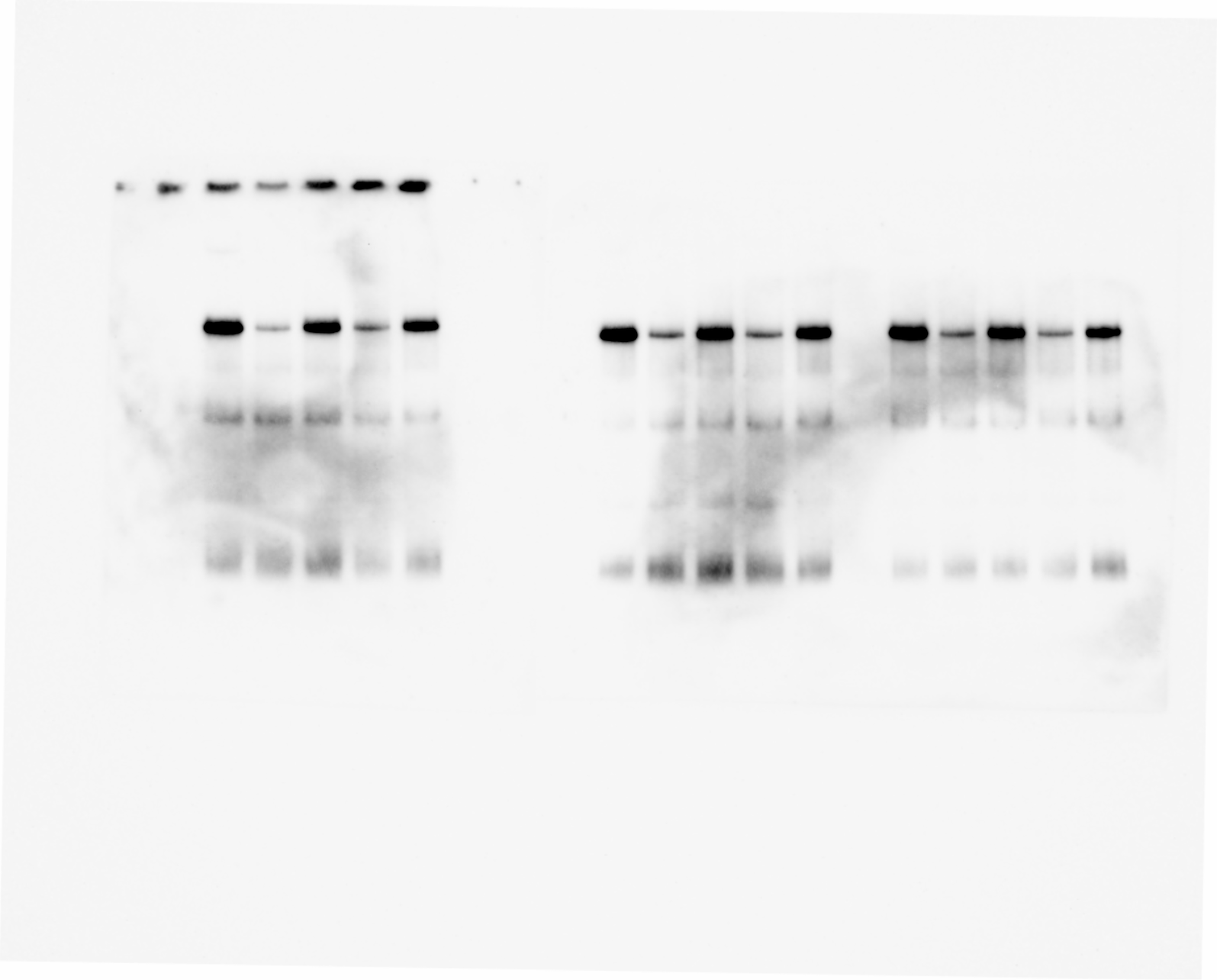

Supplement: Source data 2. [file elife-77393-data2.zip › Source data 2/WB raw data/Figure 3c/MCM3.tif]

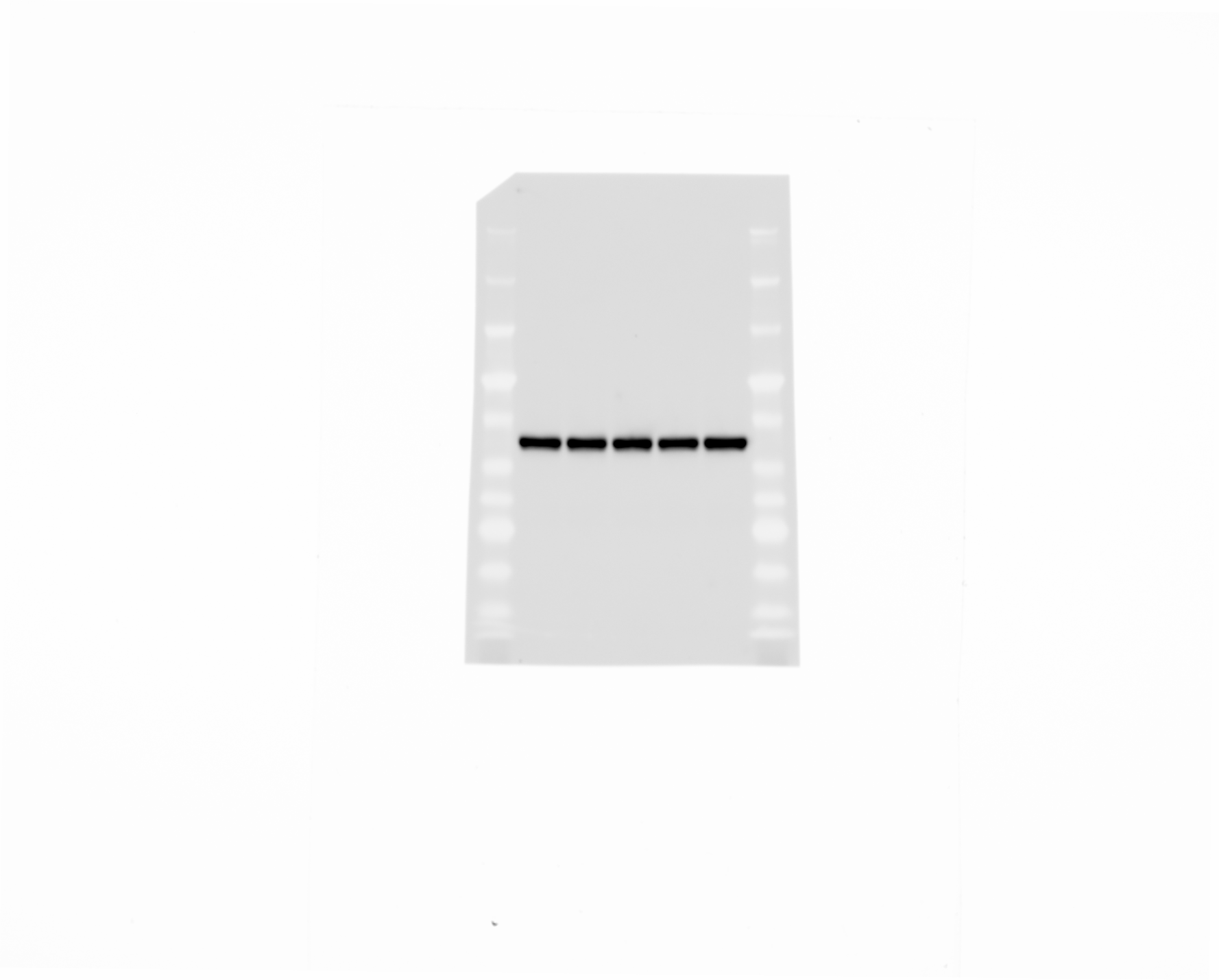

Supplement: Source data 2. [file elife-77393-data2.zip › Source data 2/WB raw data/Figure 3c/Tubulin.tif]

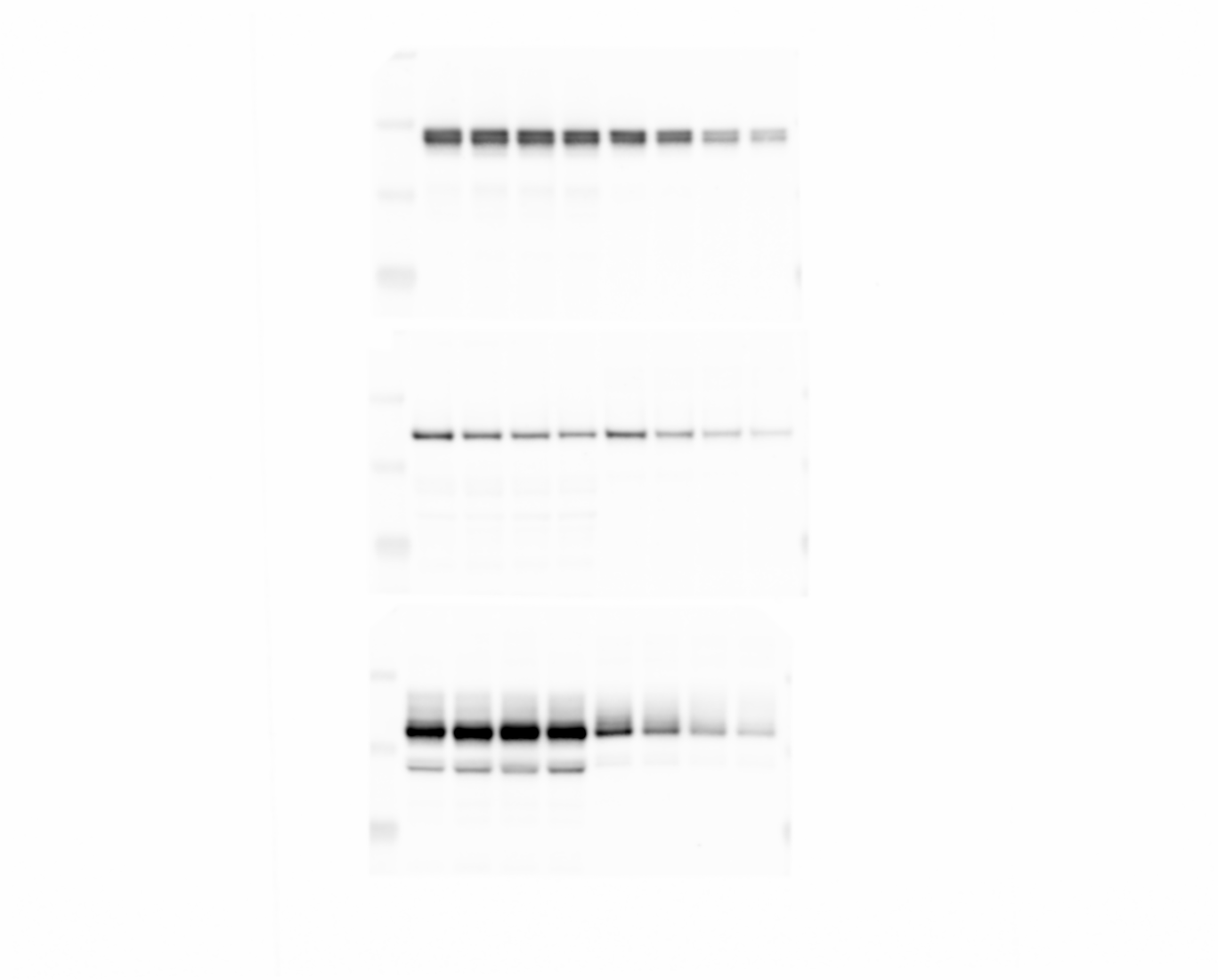

Supplement: Source data 2. [file elife-77393-data2.zip › Source data 2/WB raw data/Figure 2c/MCM4.tif]

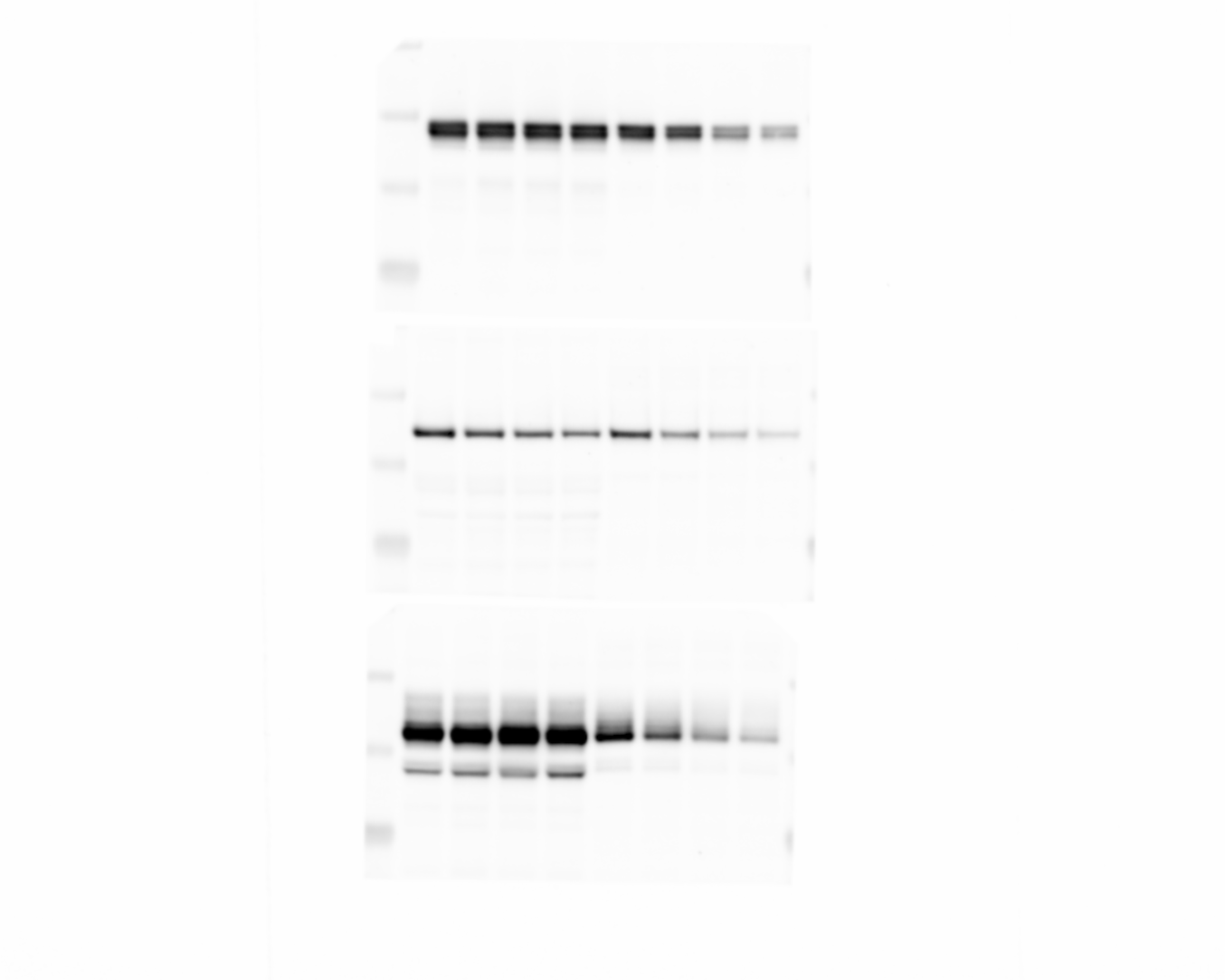

Supplement: Source data 2. [file elife-77393-data2.zip › Source data 2/WB raw data/Figure 2c/MCM2, 3.tif]

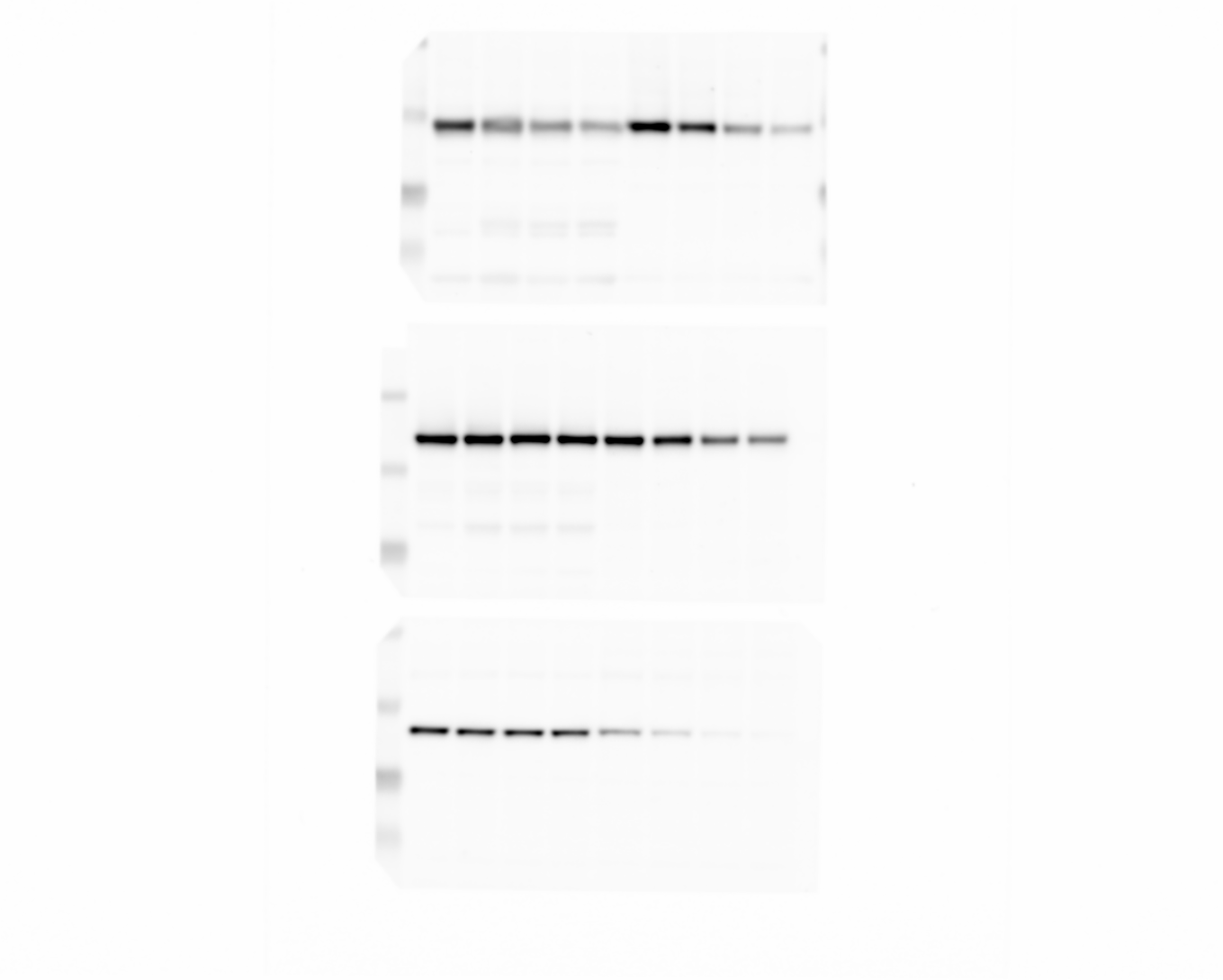

Supplement: Source data 2. [file elife-77393-data2.zip › Source data 2/WB raw data/Figure 2c/MCM5, 6, 7.tif]

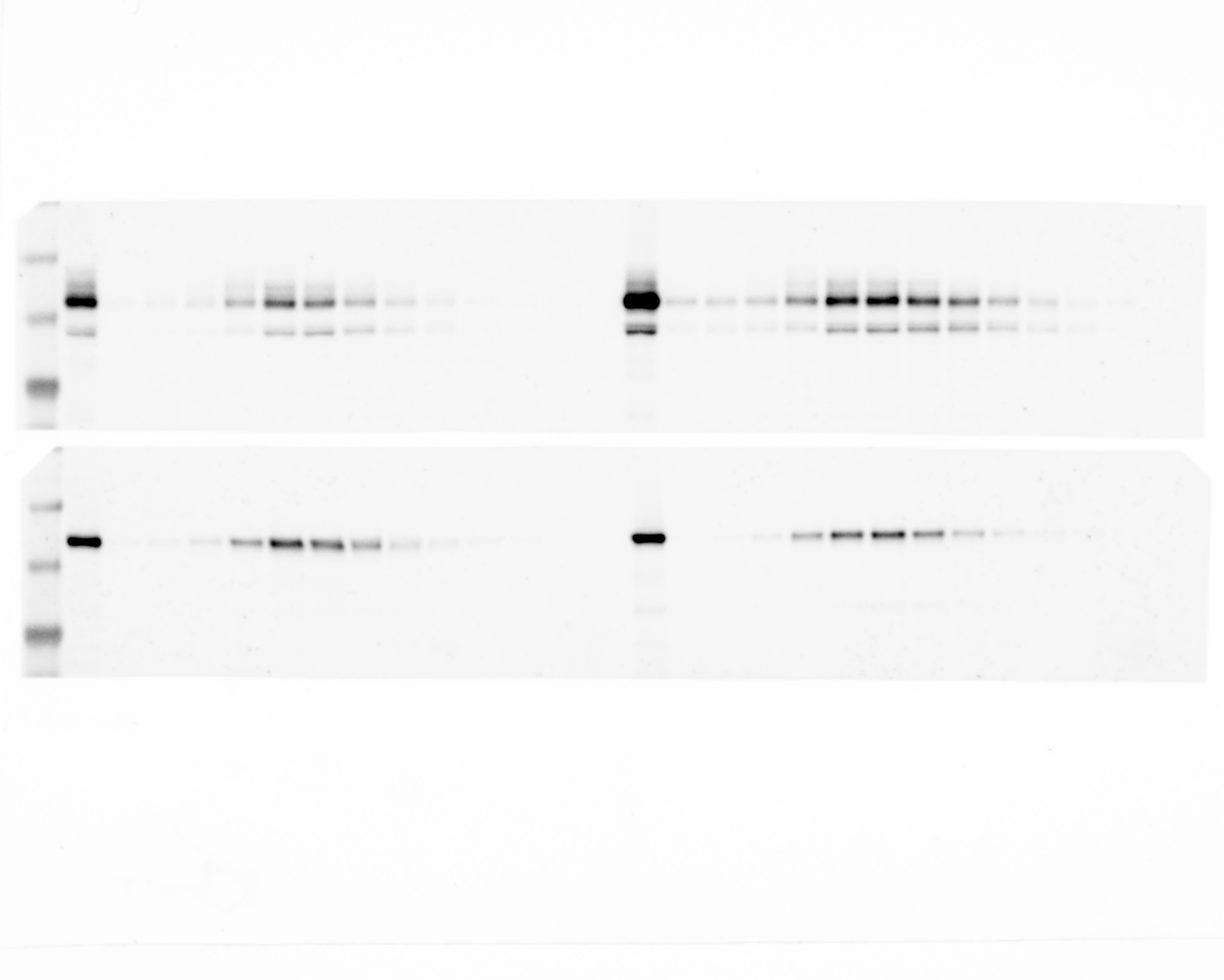

Supplement: Source data 2. [file elife-77393-data2.zip › Source data 2/WB raw data/Figure 2d/MCM4, 6.tif]

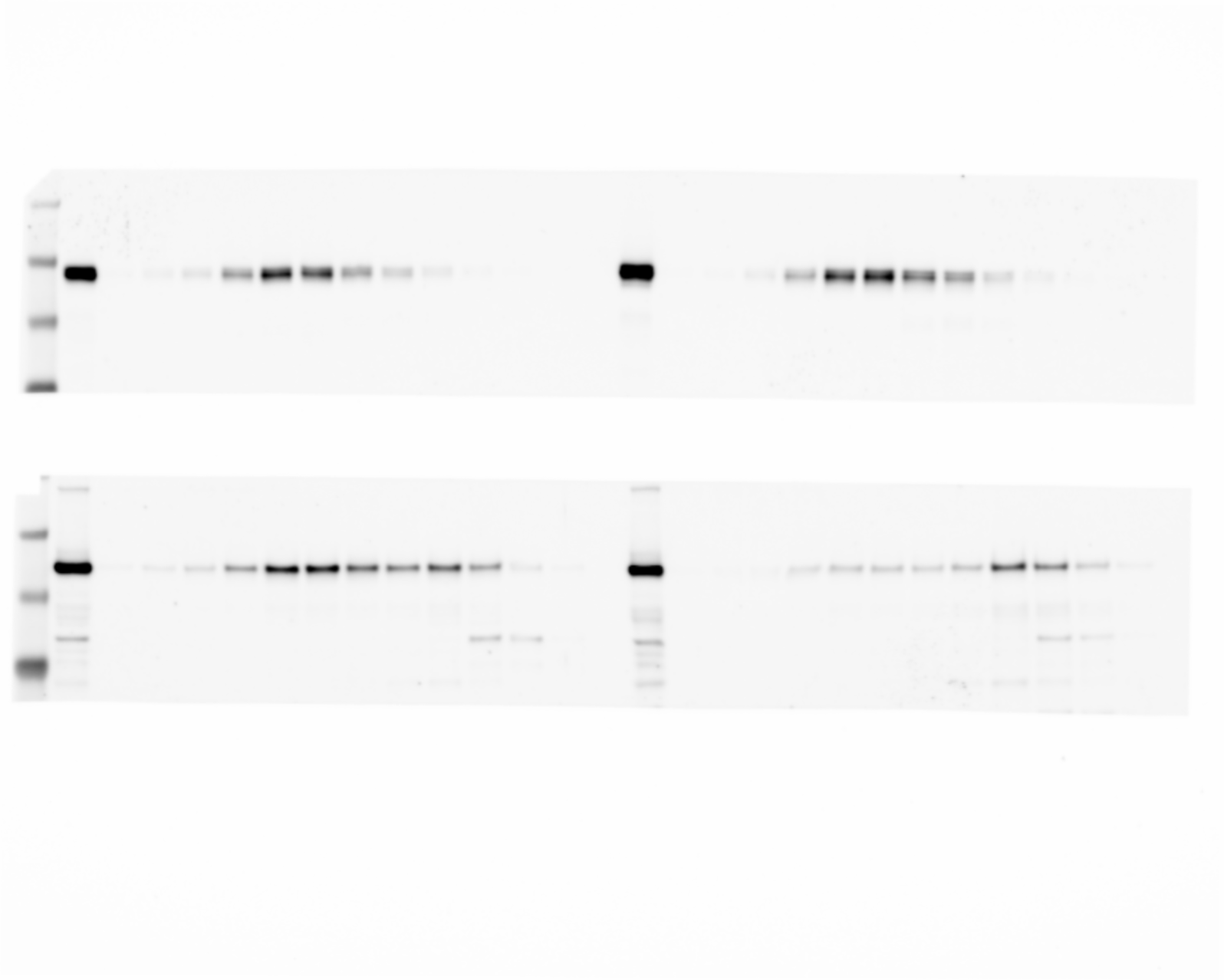

Supplement: Source data 2. [file elife-77393-data2.zip › Source data 2/WB raw data/Figure 2d/MCM3.tif]

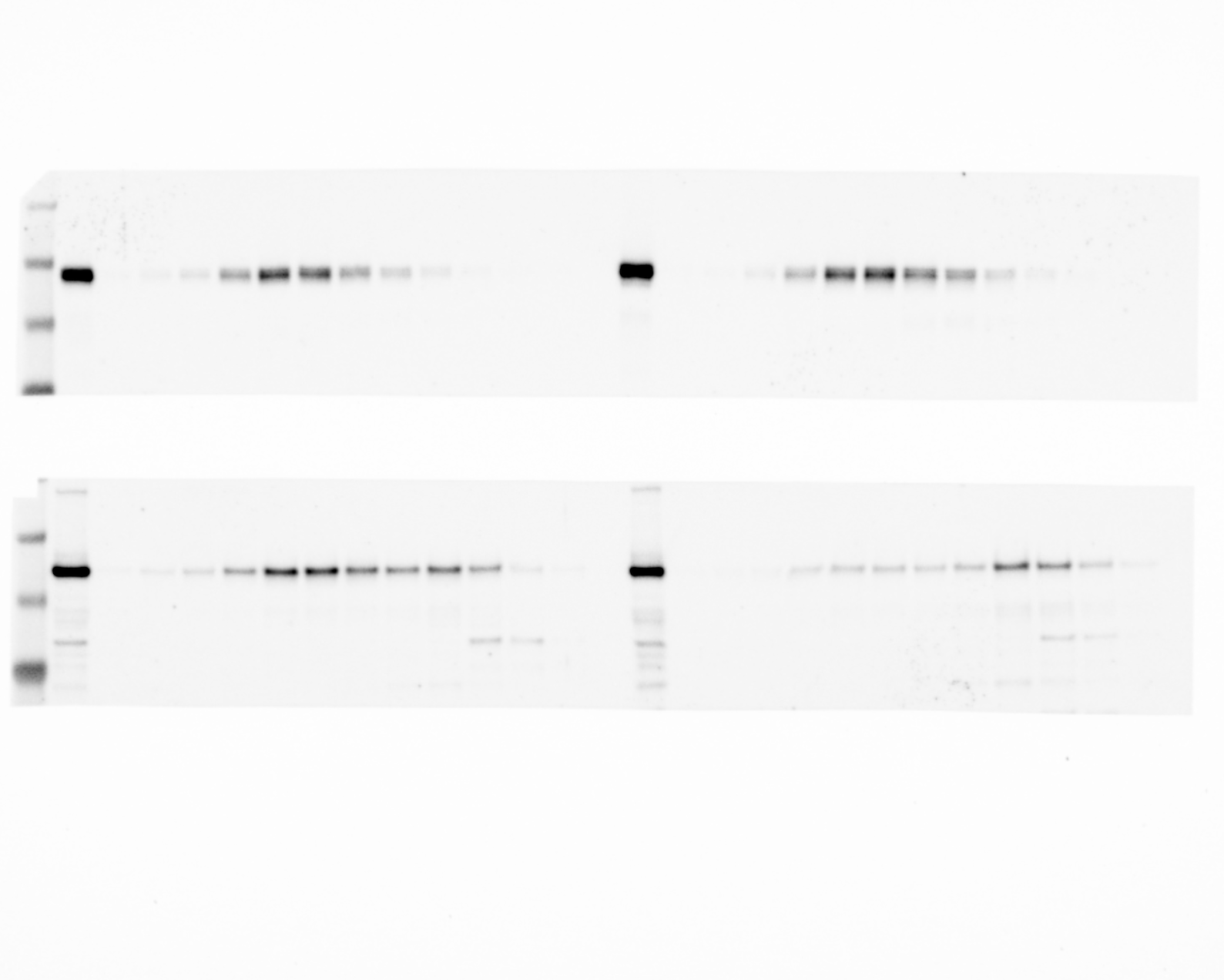

Supplement: Source data 2. [file elife-77393-data2.zip › Source data 2/WB raw data/Figure 2d/MCM2.tif]

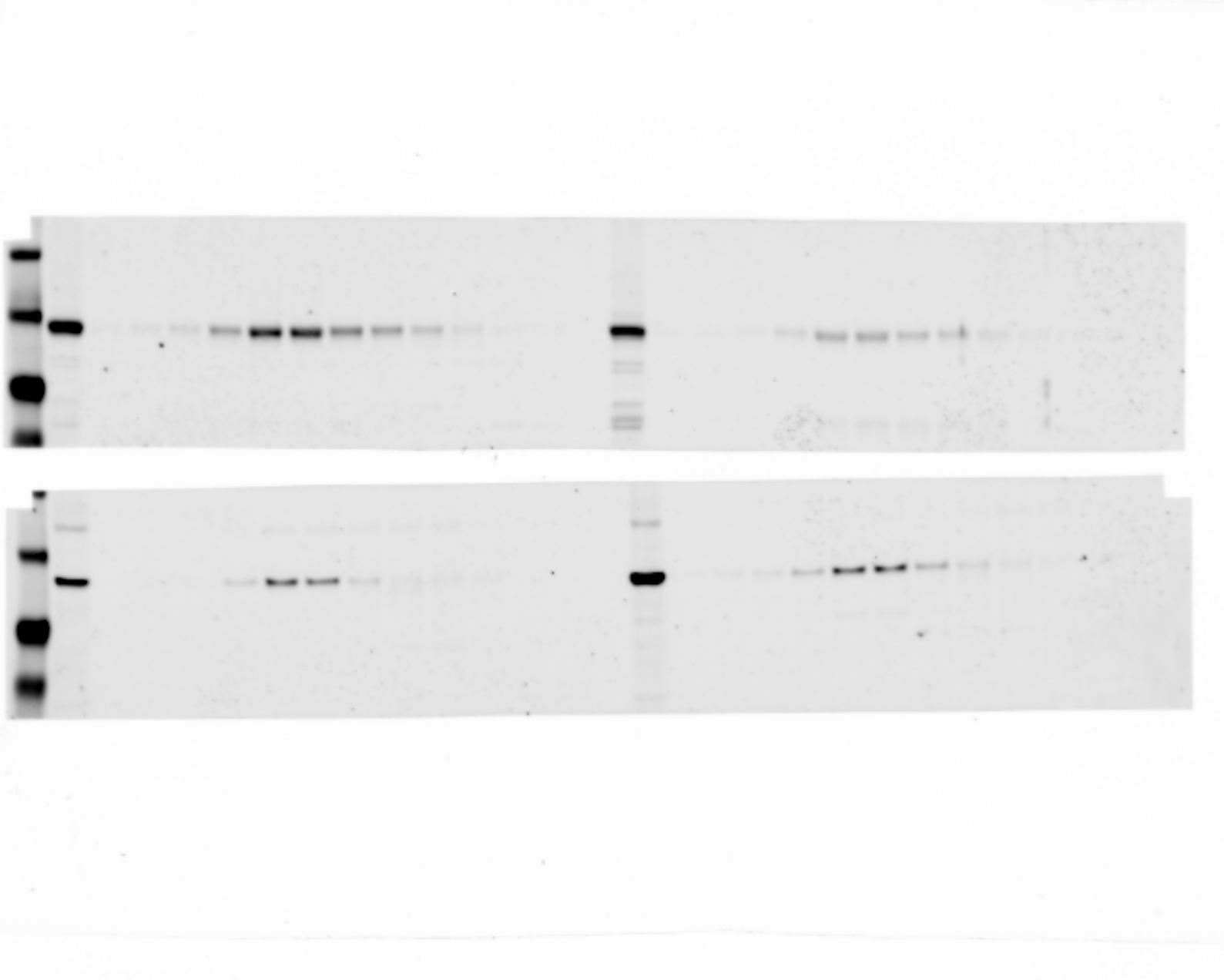

Supplement: Source data 2. [file elife-77393-data2.zip › Source data 2/WB raw data/Figure 2d/MCM5.tif]

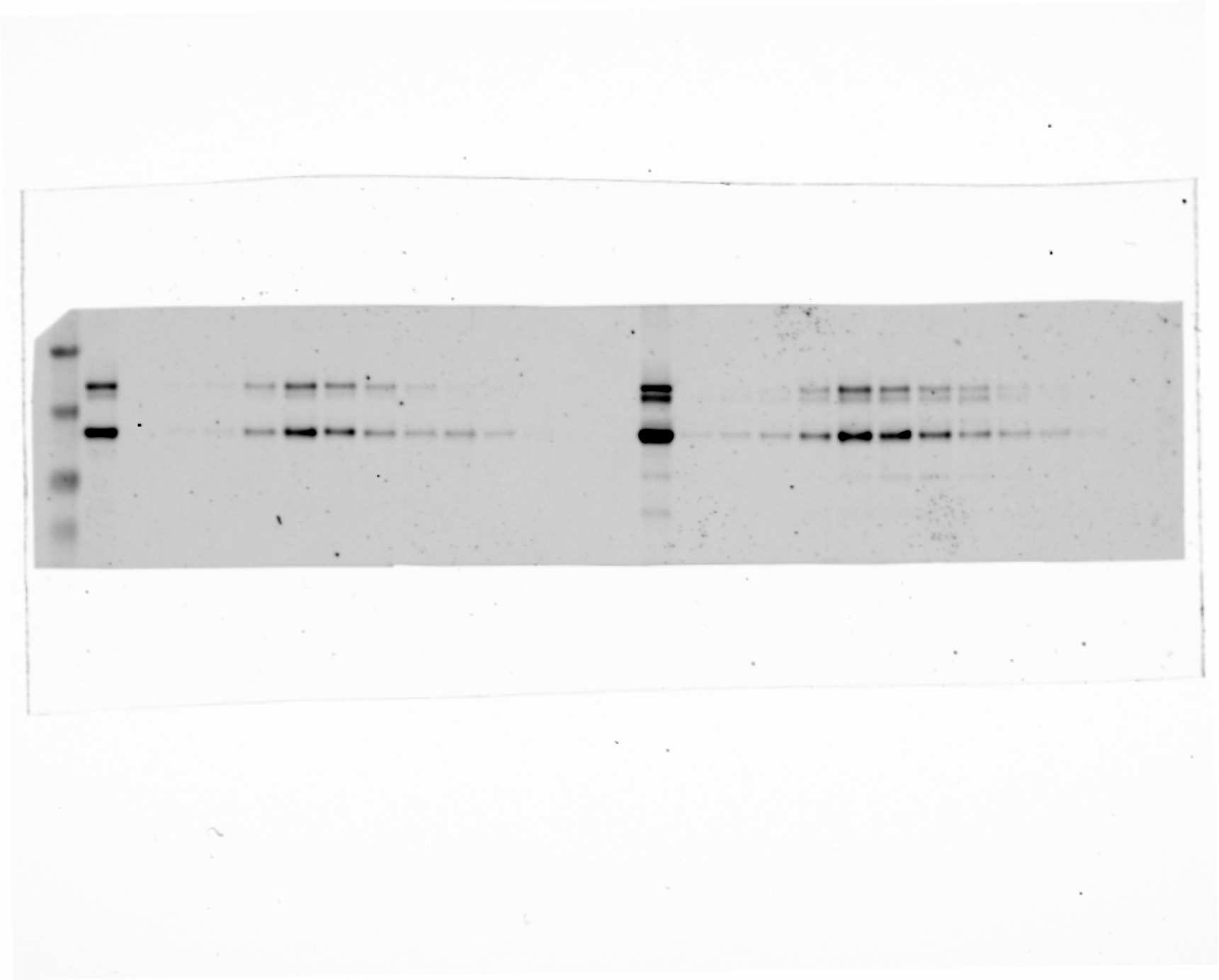

Supplement: Source data 2. [file elife-77393-data2.zip › Source data 2/WB raw data/Figure 2d/MCM7.tif]

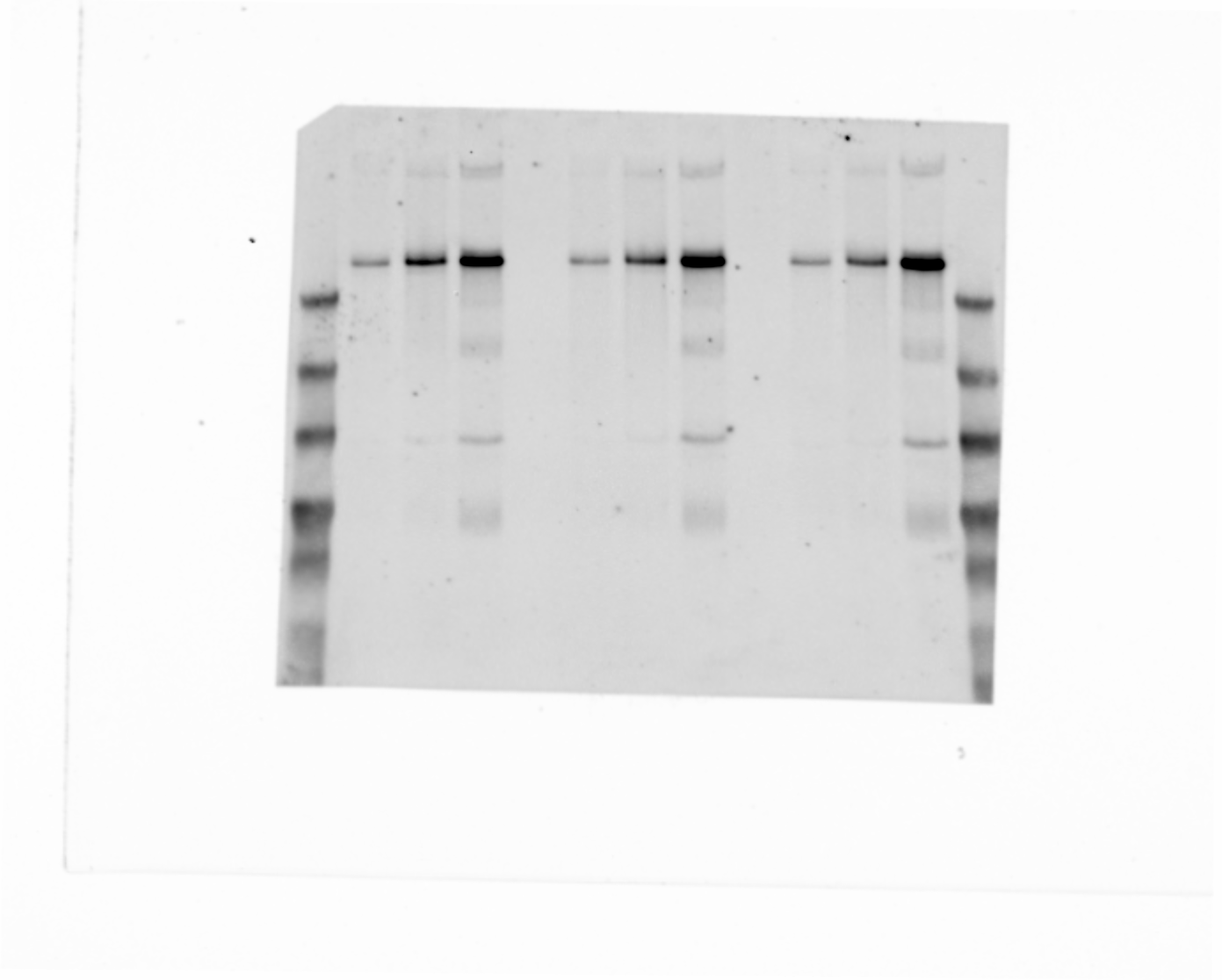

Supplement: Source data 2. [file elife-77393-data2.zip › Source data 2/WB raw data/Figure 1b/MCM3.tif]

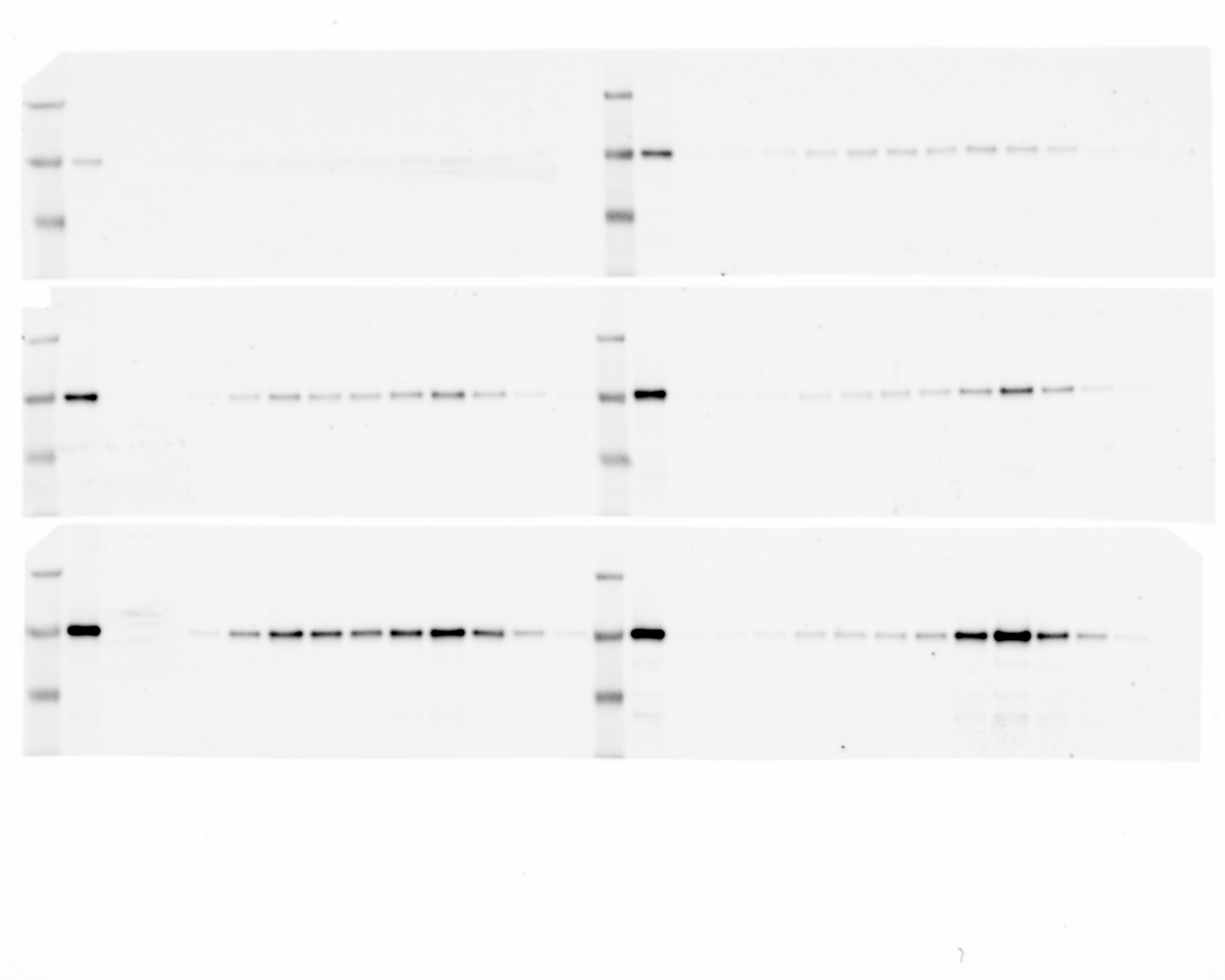

Supplement: Source data 2. [file elife-77393-data2.zip › Source data 2/WB raw data/Figure 4a/FLAG.tif]

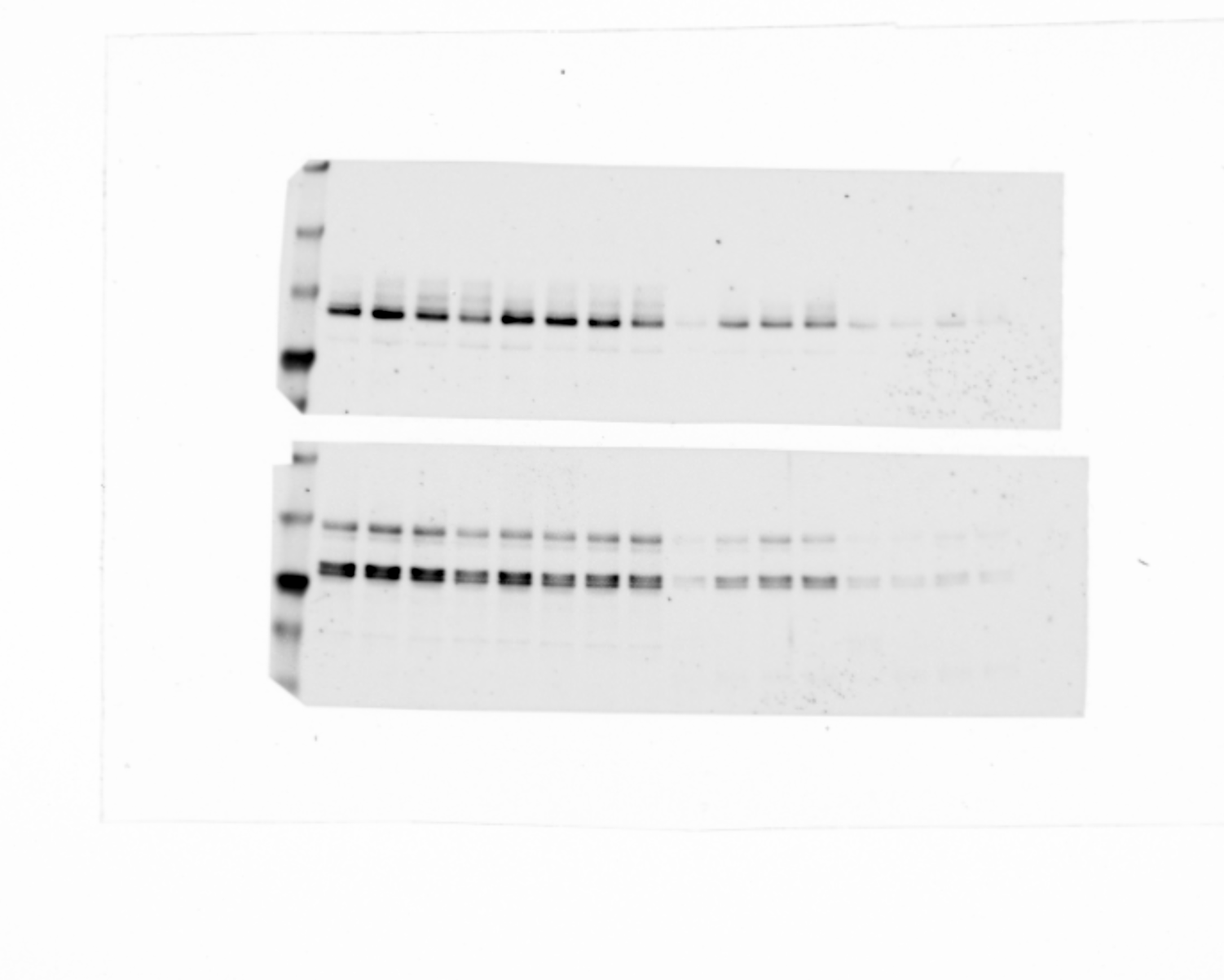

Supplement: Source data 2. [file elife-77393-data2.zip › Source data 2/WB raw data/Figure 4f/MCM4, 7.tif]

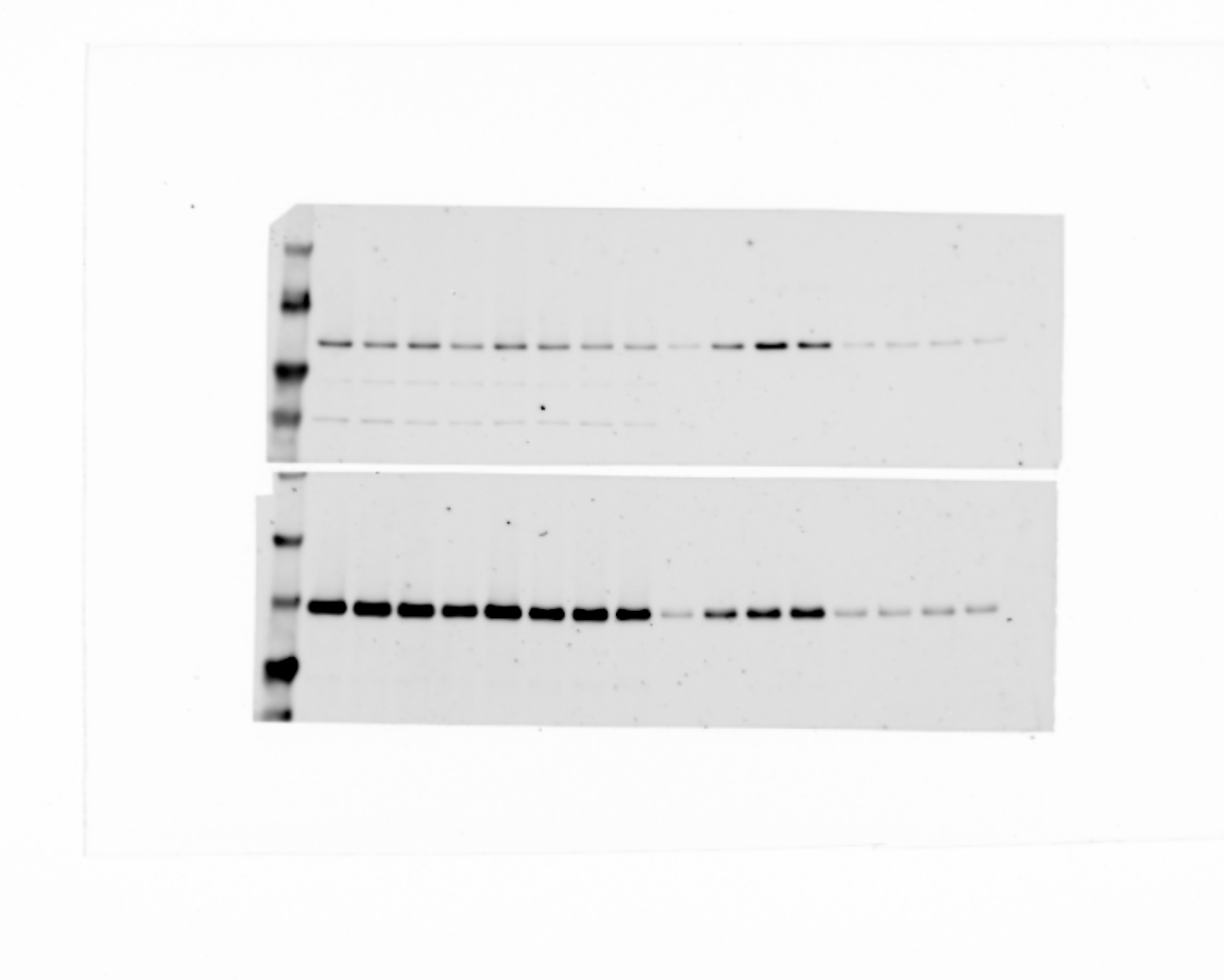

Supplement: Source data 2. [file elife-77393-data2.zip › Source data 2/WB raw data/Figure 4f/MCM5.tif]

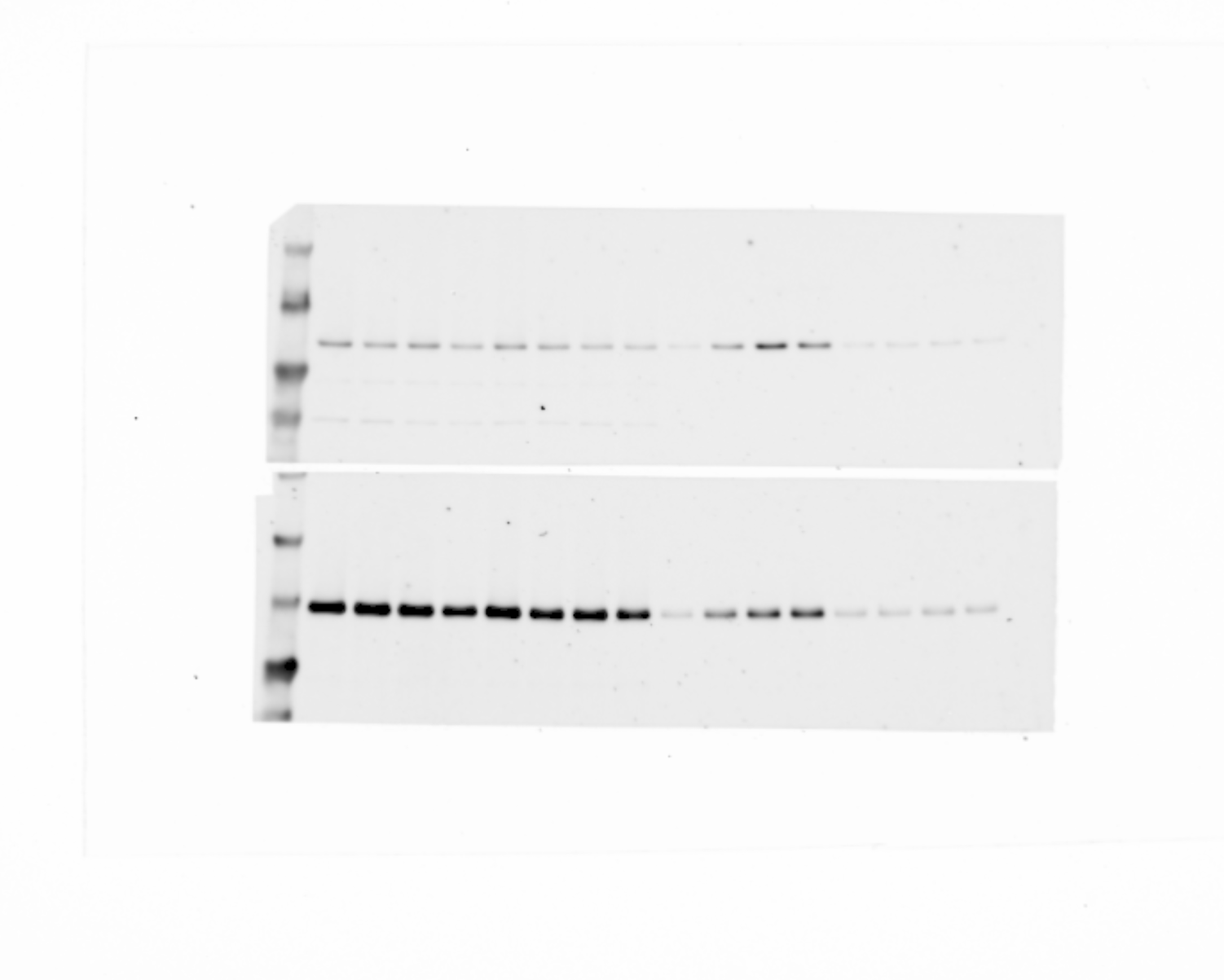

Supplement: Source data 2. [file elife-77393-data2.zip › Source data 2/WB raw data/Figure 4f/MCM6.tif]

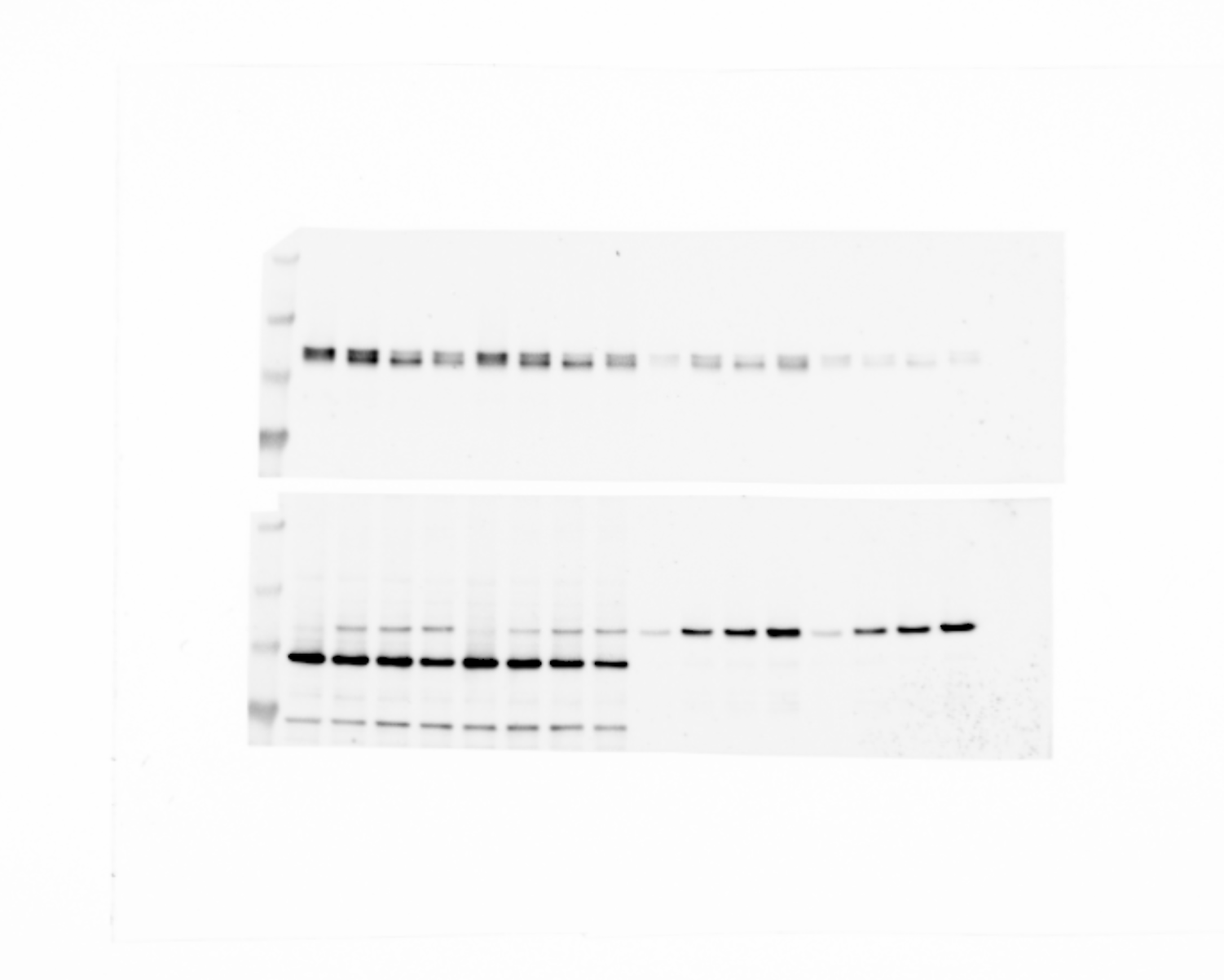

Supplement: Source data 2. [file elife-77393-data2.zip › Source data 2/WB raw data/Figure 4f/MCM2, 3.tif]

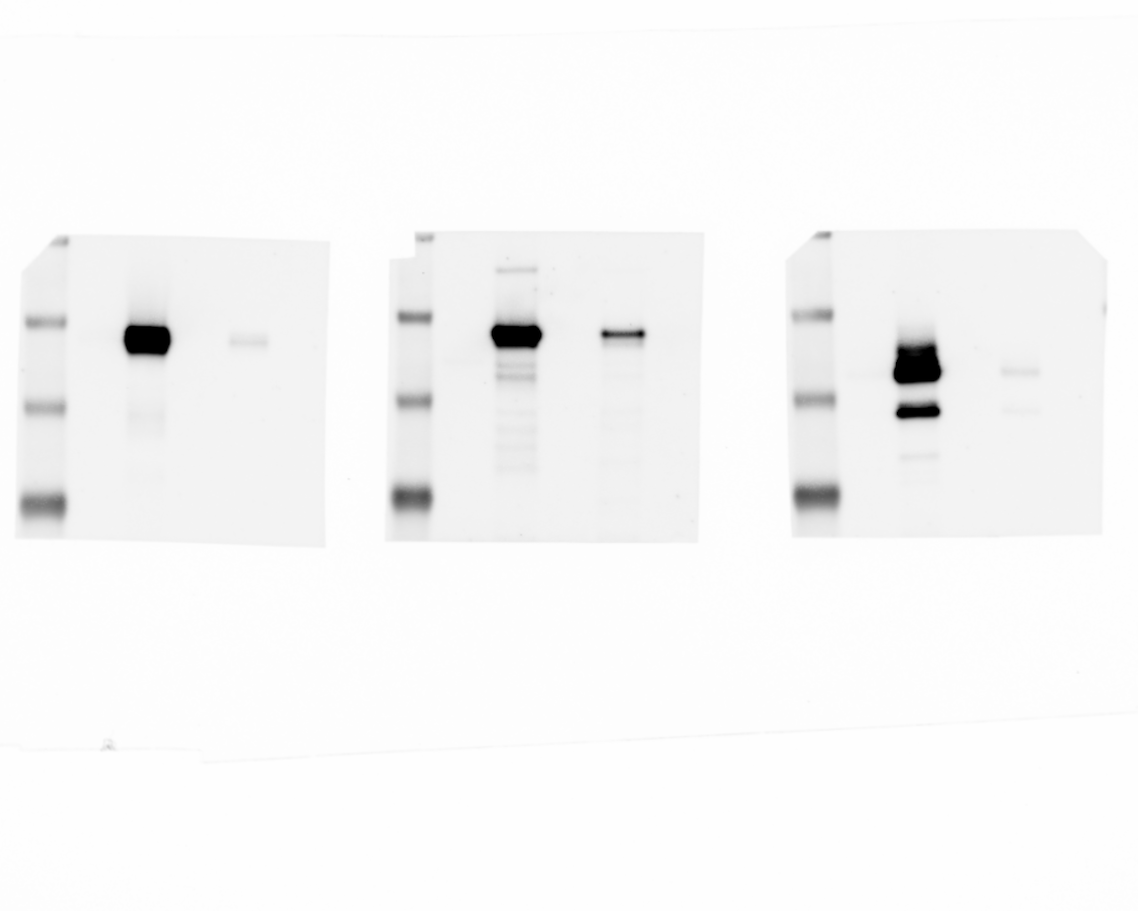

Supplement: Source data 2. [file elife-77393-data2.zip › Source data 2/WB raw data/Figure 1d/MCM3.tif]

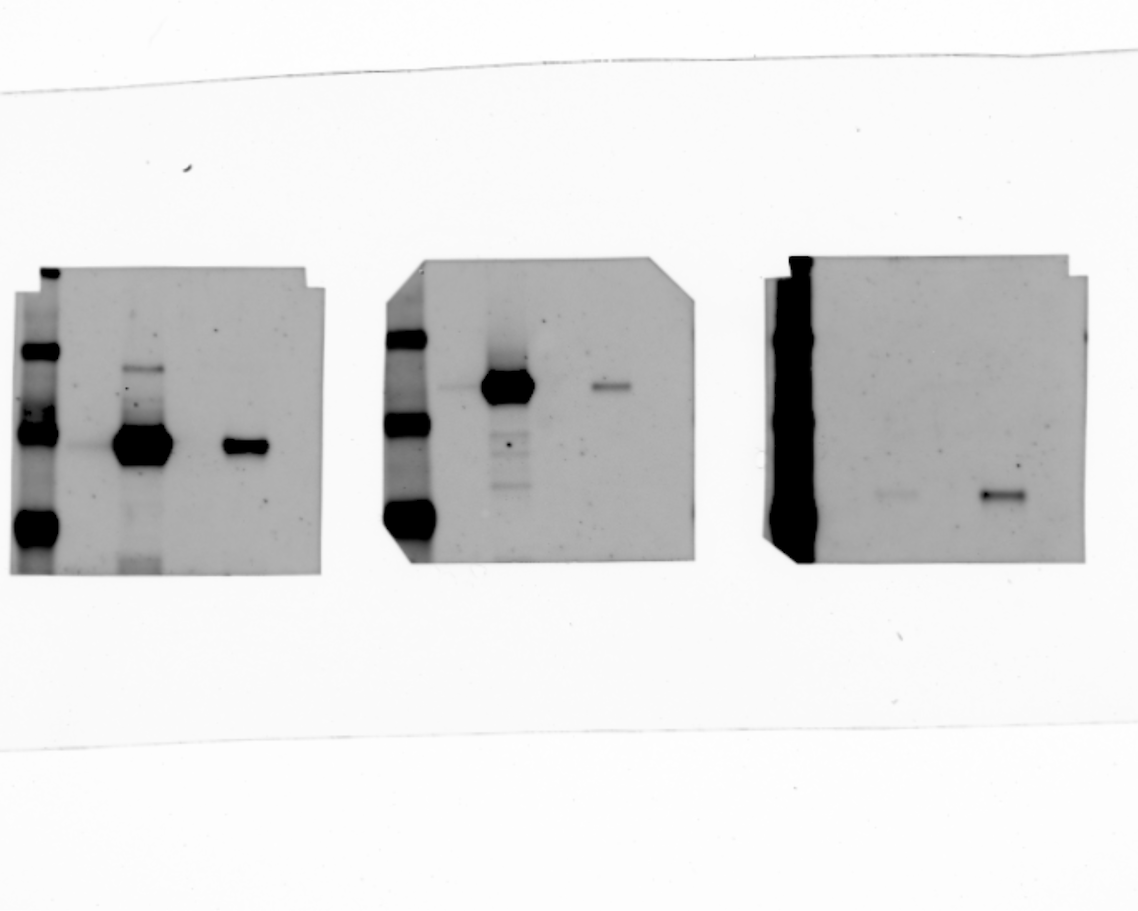

Supplement: Source data 2. [file elife-77393-data2.zip › Source data 2/WB raw data/Figure 1d/MCMBP.tif]

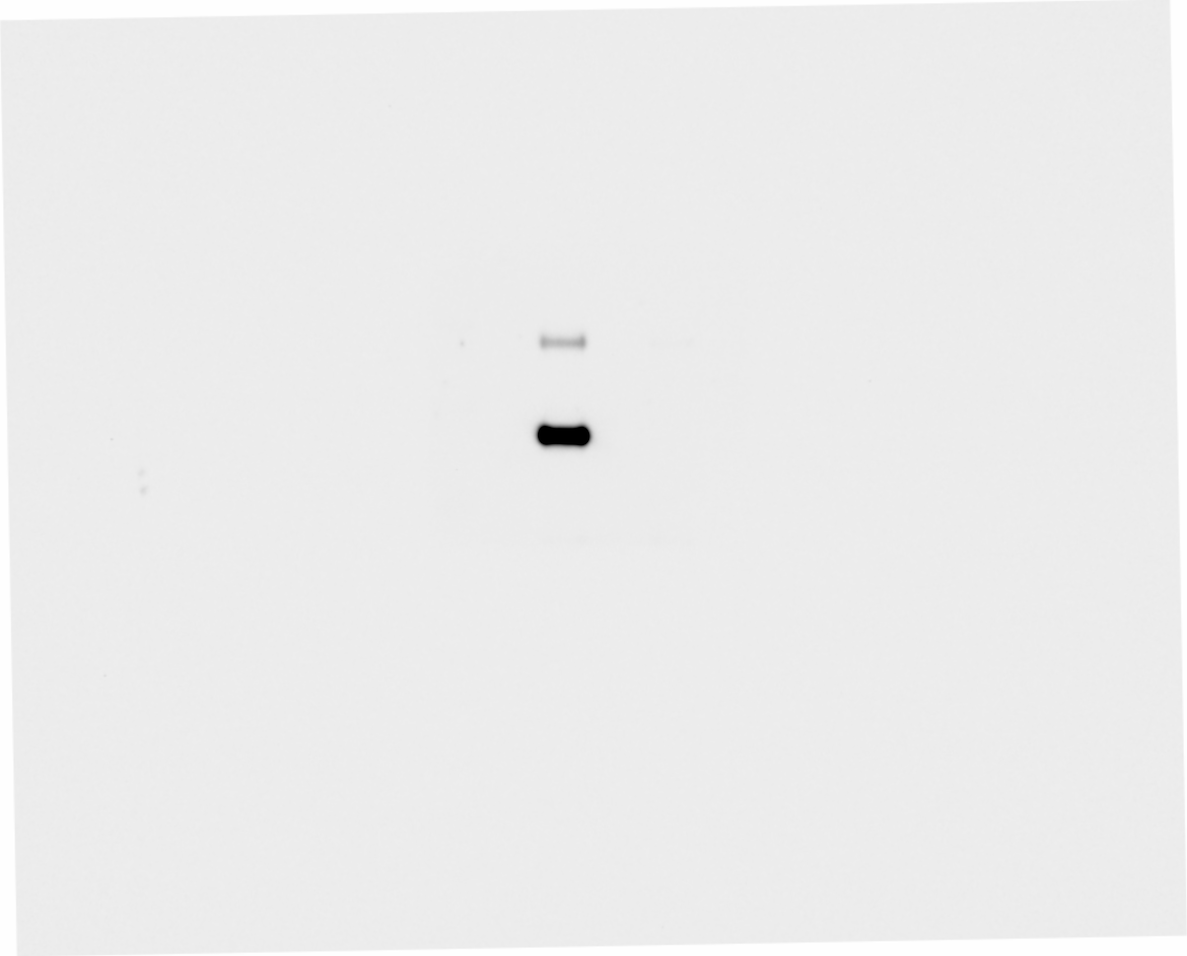

Supplement: Source data 2. [file elife-77393-data2.zip › Source data 2/WB raw data/Figure 1d/MCM7.tif]

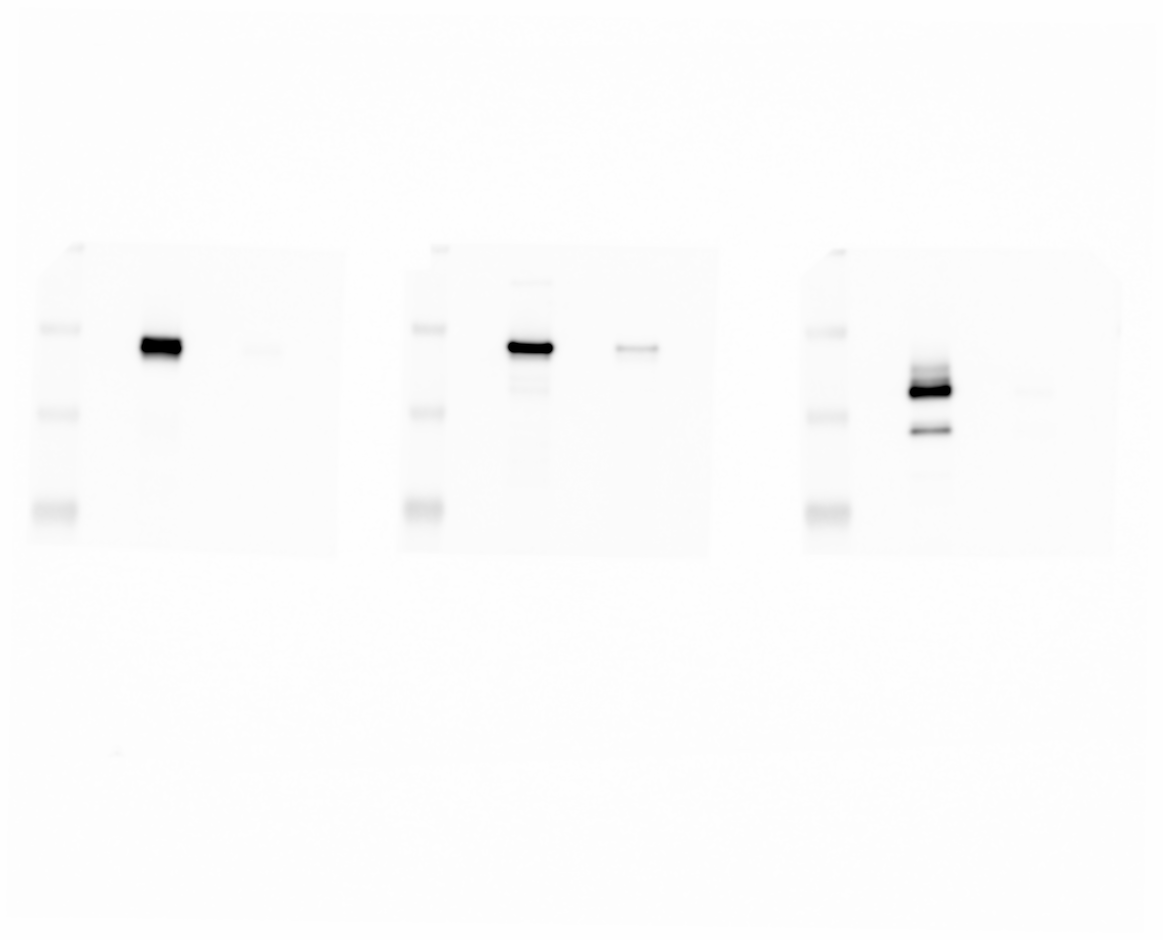

Supplement: Source data 2. [file elife-77393-data2.zip › Source data 2/WB raw data/Figure 1d/MCM2, 4.tif]

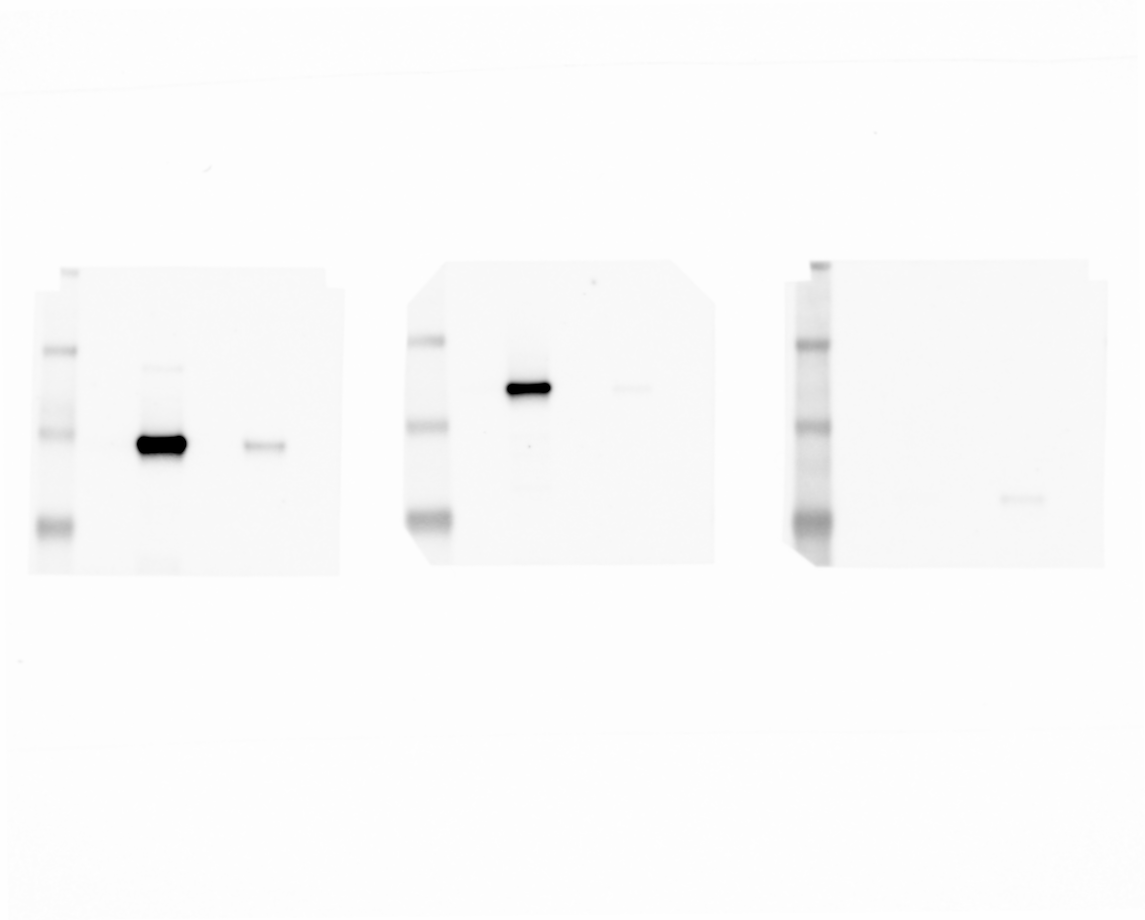

Supplement: Source data 2. [file elife-77393-data2.zip › Source data 2/WB raw data/Figure 1d/MCM5, 6.tif]

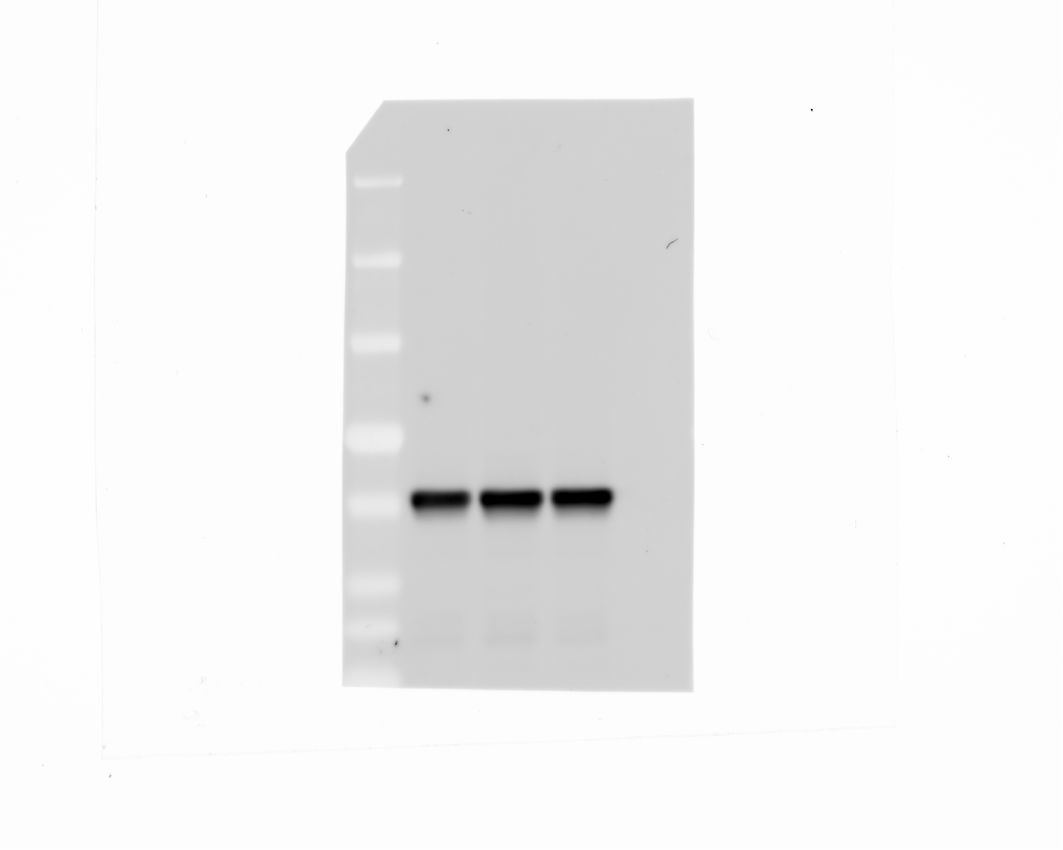

Supplement: Source data 2. [file elife-77393-data2.zip › Source data 2/WB raw data/Figure 2b/Tubulin.tif]

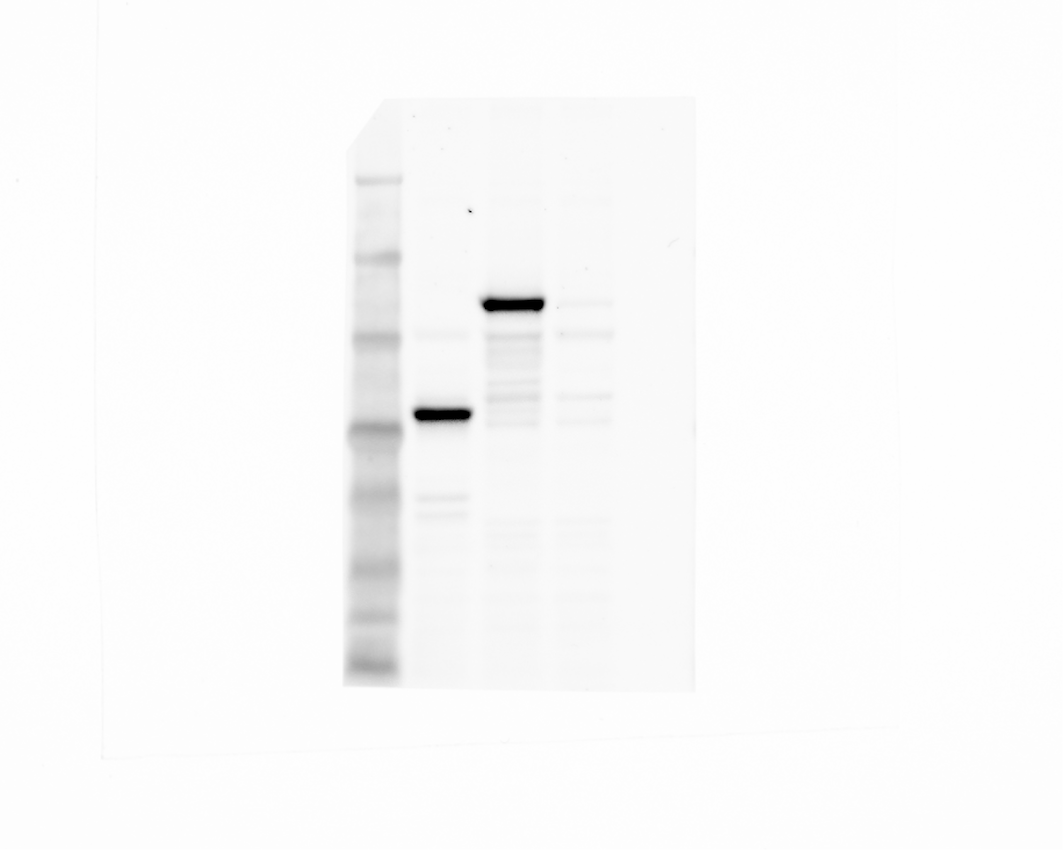

Supplement: Source data 2. [file elife-77393-data2.zip › Source data 2/WB raw data/Figure 2b/MCMBP.tif]
